# Elucidating the roles of three β-glucuronosyltransferases (GLCATs) acting on arabinogalactan-proteins using a CRISPR-Cas9 multiplexing approach in Arabidopsis

**DOI:** 10.1186/s12870-020-02420-5

**Published:** 2020-05-18

**Authors:** Yuan Zhang, Michael A. Held, Allan M. Showalter

**Affiliations:** 1 Molecular and Cellular Biology Program, Ohio University, Athens, OH 45701–2979 USA; 2 Department of Environmental & Plant Biology, Ohio University, Athens, OH 45701–2979 USA; 3 Department of Chemistry & Biochemistry, Ohio University, Athens, OH 45701–2979 USA

**Keywords:** Arabidopsis, Arabinogalactan-proteins, Calcium, Development, Glucuronic acid, Glucuronosyltransferase, Pollen, Root hairs, Seed coat mucilage, Seed germination, Siliques, Trichomes

## Abstract

**Background:**

Arabinogalactan-proteins (AGPs) are one of the most complex protein families in the plant kingdom and are present in the cell walls of all land plants. AGPs are implicated in diverse biological processes such as plant growth, development, reproduction, and stress responses. AGPs are extensively glycosylated by the addition of type II arabinogalactan (AG) polysaccharides to hydroxyproline residues in their protein cores. Glucuronic acid (GlcA) is the only negatively charged sugar added to AGPs and the functions of GlcA residues on AGPs remain to be elucidated.

**Results:**

Three members of the CAZy GT14 family (GLCAT14A-At5g39990, GLCAT14B-At5g15050, and GLCAT14C-At2g37585), which are responsible for transferring glucuronic acid (GlcA) to AGPs, were functionally characterized using a CRISPR/Cas9 gene editing approach in Arabidopsis. RNA seq and qRT-PCR data showed all three *GLCAT* genes were broadly expressed in different plant tissues, with *GLCAT14A* and *GLCAT14B* showing particularly high expression in the micropylar endosperm. Biochemical analysis of the AGPs from knock-out mutants of various *glcat* single, double, and triple mutants revealed that double and triple mutants generally had small increases of Ara and Gal and concomitant reductions of GlcA, particularly in the *glcat14a glcat14b* and *glcat14a glcat14b glcat14c* mutants. Moreover, AGPs isolated from all the *glcat* mutants displayed significant reductions in calcium binding compared to WT. Further phenotypic analyses found that the *glcat14a glcat14b* and *glcat14a glcat14b glcat14c* mutants exhibited significant delays in seed germination, reductions in root hair length, reductions in trichome branching, and accumulation of defective pollen grains. Additionally, both *glcat14b glcat14c* and *glcat14a glcat14b glcat14c* displayed significantly shorter siliques and reduced seed set. Finally, all higher-order mutants exhibited significant reductions in adherent seed coat mucilage.

**Conclusions:**

This research provides genetic evidence that GLCAT14A-C function in the transfer of GlcA to AGPs, which in turn play a role in a variety of biochemical and physiological phenotypes including calcium binding by AGPs, seed germination, root hair growth, trichome branching, pollen development, silique development, seed set, and adherent seed coat mucilage accumulation.

## Background

Arabinogalactan-proteins (AGPs) are one of the most complex protein families in plants due to the diversity of the core proteins and the heterogeneity of the glycan chains attached to these protein cores. AGPs are present on the plant cell surface including the plasma membrane via GPI anchors, cell wall, and intercellular spaces of plants. AGPs are implicated in an array of plant growth and development processes including cell expansion, somatic embryogenesis, root and stem growth, salt tolerance, hormone signaling, programmed cell death, male and female gametophyte development, and wounding/defense [1, 2]. While the diversity of AGPs is manifested by the existence of different protein domains in the protein core, multiple glycans (AG chains) decorating each protein core, and the heterogeneity of glycosylation, they are unified by a common structure. Specifically, AGPs are *O*-linked glycoproteins characterized by the presence of type II (β-1,3 and β-1,6) AGs attached to hydroxyproline (Hyp) residues present in the protein core. More simply, the Hyp residues in AGP core proteins are modified by the addition of β-1,3-galactose sugar backbones, which are further modified by the addition of multiple, branching β-1,6 galactose side chains, which are further modified by the addition of arabinose (Ara) residues and less extensively by other sugars, such as rhamnose (Rha), fucose (Fuc), xylose (Xyl) and glucuronic acid (GlcA) [3, 4].

Several glycosyltransferases (GTs), each of which is responsible for transferring individual monosaccharides to synthesize the AGP glycans, are localized mainly in the Golgi. They were isolated and identified using both biochemical and bioinformatics approaches. Previously, our lab discovered five β-1,3-galactosyltransferases (GALT2, GALT3, GALT4, GALT5, and GALT6) [5, 6]. Three additional GALTs were subsequently discovered namely HPGT1, HPGT2, and HPGT3 [7]. All eight enzymes are from the Carbohydrate Active Enzyme (CAZy) GT31 family and function in catalyzing the addition of the first sugar, galactose, onto hydroxyproline residues contained in the AGP core proteins in Arabidopsis*.* Single genetic mutants of the GALT family showed subtle or no phenotypic changes compared to wild type (control) plants. However, the *hpgt* triple mutants exhibited longer root hairs, smaller leaves, and reductions in plant height and seed set [7]. In contrast, the *galt2 galt5* double mutant demonstrated phenotypes such as shorter root length and root swelling phenotype under salt stress condition, as well as a reduction in seed coat mucilage [8]. Another β-1,3-galactosyltransferase named KNS4/GALT14, which is also found in the CAZy GT31 family, was recently characterized in Arabidopsis [9]. Mutant analyses found that *ksn4* had an irregular and collapsed pollen phenotype, which was due to abnormal formation of the exine layer in the developing microspore [9]. AtGALT29A and AtGALT31A from the CAZy GT29 and GT31 families, respectively, represent two β-1,6-galactosyltransferases that were also identified in Arabidopsis [10]. In addition, a β-arabinofuranosyltransferase, RAY1, was also characterized in Arabidopsis. The *ray1* mutant showed a reduction of arabinofuranose (Ara*f*) in its AGPs [11]. Although heterologous expression of RAY1 in tobacco microsomes demonstrated β-Ara*f* activity, β-1,3-linked Ara*f* has not been found in plants. Moreover, two α-1,2–fucosyltransferases, AtFUT4 and AtFUT6, were also identified through heterologous expression in tobacco BY2 cells [12]. AtFUT4 and AtFUT6 were able to catalyze the transfer of fucose to AGPs in vitro [12]. Further research found that double mutants of *fut4 fut6* contained no fucose and exhibited reduced root length under salt stress conditions compared to wild-type (WT) [13]. Finally, three β-glucuronosyltransferases (GLCATs) in the CAZy GT14 family, *GLCAT14A*, *GLCAT14B*, and *GLCAT14C* were identified through co-expression analyses with AGPs in Arabidopsis and verified by heterologous expression and an in vitro enzyme assay [14, 15]. So far only GLCAT14A has been functionally characterized to some extent by the use of mutants [14]; however, no such work has been done for GLCAT14B and GLCAT14C.

Due to gene redundancy among the GT families, a comprehensive understanding of the functions of the diverse sugars decorating the AGP protein core requires the generation of higher-order mutants in order to substantially reduce or eliminate addition of a particular sugar residue and observe its functional consequences. The traditional (T-DNA based) method of generating multiple gene knockout mutants is both labor-intensive and time-consuming. Moreover, genetic linkage, which is commonly seen in GTs and cell wall families, also makes it more challenging to generate higher-order mutants by conventional crossing method.

Consequently, we have chosen to utilize CRISPR-Cas9 gene editing technology to address this issue of gene redundancy in the GT families. CRISPR-Cas9 allows for the production of mutations in multiple genes in a single transformation event by the design of multiple guide RNAs (gRNAs) targeting multiple GT genes. Here we report on the successful use of CRISPR-Cas9 gene editing technology to target three *GLCAT* genes, known to be involved in the transfer of GlcA residues to AGPs as a test case. The resulting mutants not only demonstrate the feasibility of this approach, but also provide new evidence for the function of GlcA residues in AGPs with respect to plant growth and development.

## Results

### GLCAT14A, GLCAT14B, and GLCAT14C belong to the CAZy GT14 family

This study focused on functional characterization of GLCAT14A (At5g39990), GLCAT14B (At5g15050), and GLCAT14C (At2g37585) found in the CAZy GT14 family, which includes 11 proteins in Arabidopsis. The GT14 family was identified by a branch domain (Pfam 02485), which is named as a GLCAT domain [16]. Following heterologous expression of these 11 Arabidopsis GT14 members in *Pichia pastoris*, only three members (GLCAT14A, GLCAT14B, and GLCAT14C) exhibited GlcA transferase activity when using UDP-^14^[C]-GlcA as the sugar donor and AGP substrate acceptors [14, 15]. While all three enzymes demonstrated GlcA transferase activity with β-1,3 galactan and β-1,6 galactan AGP substrate acceptors, GLCAT14A and GLCAT14B preferred the former acceptor, while GLCAT14C preferred the later acceptor [14, 15]. Furthermore, co-expression analysis by GeneCAT found that GLCAT14A was co-expressed with AtGALT31A [14, 17].

Among the three *GLCATs*, *GLCAT14A* and *GLCAT14B* share 73.3% amino acid similarity, but only share 43.4 and 48.3% amino acid similarity with *GLCAT14C*, respectively (Additional file 1: Figure S1). *GLCAT14A* and *GLCAT14B* are both located on chromosome 5, whereas *GLCAT14C* is found on chromosome 2.

### Expression profiles of *GLCAT14A*, *GLCAT14B*, and *GLCAT14C*

**Fig. 1:**
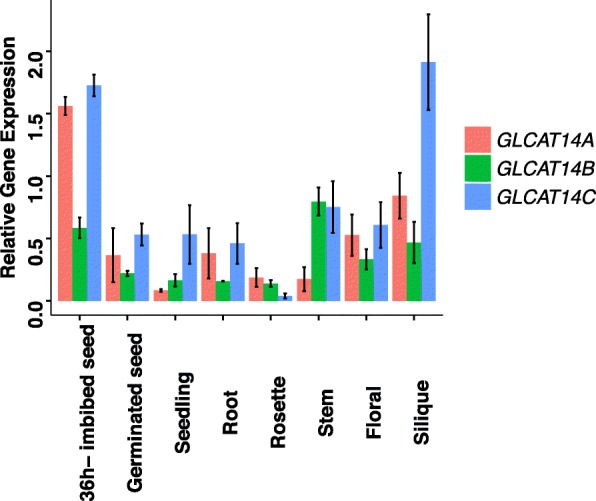
Relative gene expression of *GLCAT14A*, *GLCAT14B*, *and GLCAT14C* in different organs and developmental stages of Arabidopsis (*Col-0*) as determined by qRT-PCR. Transcript levels were normalized to the mean of one reference gene, the Arabidopsis Actin 2 gene, *AtACT2*. Averages of three biological replicates ± SE are shown

Our qRT-PCR and publicly available RNA-seq data from the Klepikova Atlas [18] were used to examine expression levels of the three GLCATs (Fig. 1, Additional file 1: Figure S2). Both the qRT-PCR and RNA-seq data showed that *GLCAT14A* and *GLCAT14C* were expressed at a higher level than *GLCAT14B* in most tissues. In addition, qRT-PCR results showed that all three GLCATs exhibited high expression in 36 h water-imbibed seeds and siliques than other tissues. *GLCAT14B* also showed a high expression in stems. Publicly available RNA-seq data additionally showed that *GLCAT14A* exhibited higher expression in seedling roots, leaf petioles, and siliques, *GLCAT14B* was mostly expressed in mature leaves, internodes, and leaf petioles, and GLCAT14C was highly expressed in flowers, seeds and siliques. The high expression values for both GLCAT14A and GLCAT14C in siliques and for GLCAT14B in internodes based on RNA-seq data were consistent with our qRT-PCR results. In addition, transcriptome data from developing seeds as displayed in the eFP browser [19] found that *GLCAT14A* and *GLCAT14B* were very highly expressed in the micropylar endosperm at the preglobular and globular stages of seed development in contrast to GLCAT14C which was not expressed at this location (Additional file 1: Figure S3). Both *GLCAT14A* and *GLCAT14C* also had moderate expression in the seed coat, whereas *GLCAT14B* was exclusively found in the micropylar endosperm. Given the high expression levels in micropylar endosperm for *GLCAT14A* and *GLCAT14B*, and in water-imbibed seeds for *GLCAT14A* and *GLCAT14C* as shown by qRT-PCR, the three *GLCATs* were speculated to play a role in seed development and germination.

### Generation of *GLCAT* higher-order mutants using a CRISPR-Cas9 multiplexing approach

To date, only *GLCAT14A* in the Arabidopsis GT14 family was subjected to mutant analysis and partial characterization. Two allelic T-DNA insertion mutants for *GLCAT14A*, named *glcat14a-1* and *glcat14a-2*, were obtained and showed a reduced level of β-GLCAT activity as well as a 12% increase of Gal and 12% decrease of Ara in seedling AGPs. Physiologically, both allelic mutants displayed a 30% increase in root length and a 30% increase of hypocotyl length when grown in the dark [14]. However, as *GLCAT14A* and *GLCAT14B* likely redundant, a single genetic mutant such as *glcat14a* is often not sufficient to uncover fully the biological functions of GlcA residues. Furthermore, it is not ideal to use a traditional crossing method to create a double mutant for two genetically linked genes such as *GLCAT14A* and *GLCAT14B*. Therefore, this study utilized a multiplexing CRISPR-Cas9 gene editing strategy to create and examine higher-order knockout mutants for all three *GLCATs* simultaneously.

**Fig. 2:**
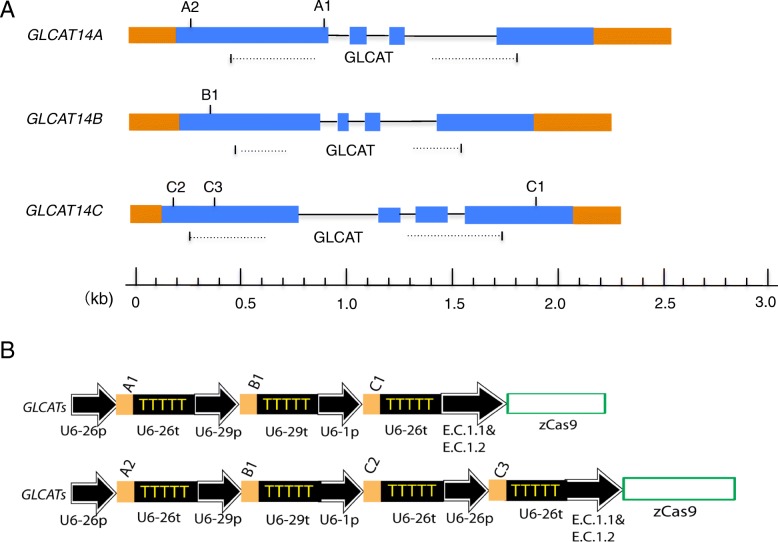
Schematic diagrams of guide RNA (gRNA) targeting sites and CRISPR-Cas9 multiplexing constructs to target three genes encoding glucuronic acid transferases (GLCATs)*.***A.** Two sites were chosen to target *GLCAT14A* (A1 and A2), one site was chosen to target *GLCAT14B* (B1), and three sites were chosen to target *GLCAT14C* (C1, C2, and C3). Online software CRISPR-P 2.0 (http://crispr.hzau.cn/cgi-bin/CRISPR2/CRISPR) was used for designing all gRNAs. Pfam domain predictions: Pf02485 identified the glucuronosyltransferase (GLCAT) domain (http://www.sanger.ac.uk/Software/Pfam/). **B. (1)** Three gRNAs (A1, B1, and C1 in Figure 2A) were assembled head-to-tail with each gRNA regulated by an individual promoter and terminator to target the three *GLCATs* (*GLCAT14A*, *GLCAT14B*, and *GLCAT14C*). **(2)** Four gRNAs (A2, B1, C1, and C2 in Figure 2A) were assembled head-to-tail to target three *GLCATs* (*GLCAT14A*, *GLCAT14B*, and *GLCAT14C*). The two constructs [**(1)** and **(2)**] were cloned into the pHEE401E plasmid vector engineered by Wang et al., 2015b), which contains a maize codon-optimized Cas9 (zCas9) gene driven by an Arabidopsis egg-cell specific promoter (E.C1.1) fused with an egg-cell specific enhancer (E.C1.2)

Six gRNAs (A1, A2, B1, C1, C2, and C3) were designed to target *GLCAT14A*, *GLCAT14B*, *and GLCAT14C* at different loci (Fig. 2A), and they were assembled into two CRISPR/Cas9 multiplexing constructs using the pHEE401E binary vector, which also contains a *Zea mays* codon-optimized *zCas9* gene under the control of an Arabidopsis egg-cell specific promoter (E.C1.1) fused with an egg-cell specific enhancer (E.C1.2) [20]. Previous studies using pHEE401E successfully created higher-order homozygous CRISPR mutants in a single generation in Arabidopsis [21]. With our two CRISPR constructs, a number of single and double *glcat* mutants were created, and we chose one Cas9-free line for each of the resulting mutants: *glcat14b*, *glcat14c*, *glcat14a glcat14b*, and *glcat14b glcat14c* for functional characterization. In addition, one *glcat14a glcat14b glcat14c* triple *glcat* CRISPR mutant line was identified and used for further characterization.

**Fig. 3:**
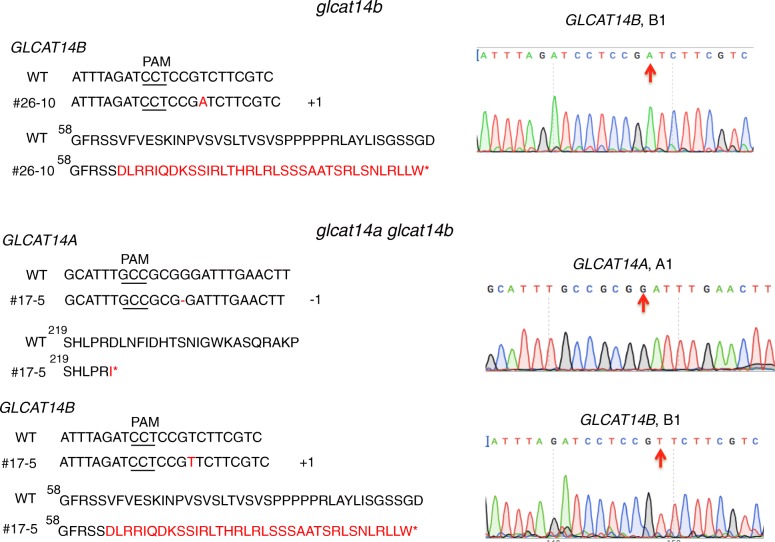
Sequencing results of the two target sites (A1 and B1) of the *glcat14a glcat14b* double mutant (line #17–5). *GLCAT14A* contained a 1 bp deletion, which resulted in a pre-mature stop codon; *GLCAT14B* contained a 1 bp insertion, which resulted in a pre-mature stop codon in its protein sequence. Sequences of the mutation sites are indicated by red arrows on the chromatograms on the right

**Fig. 4:**
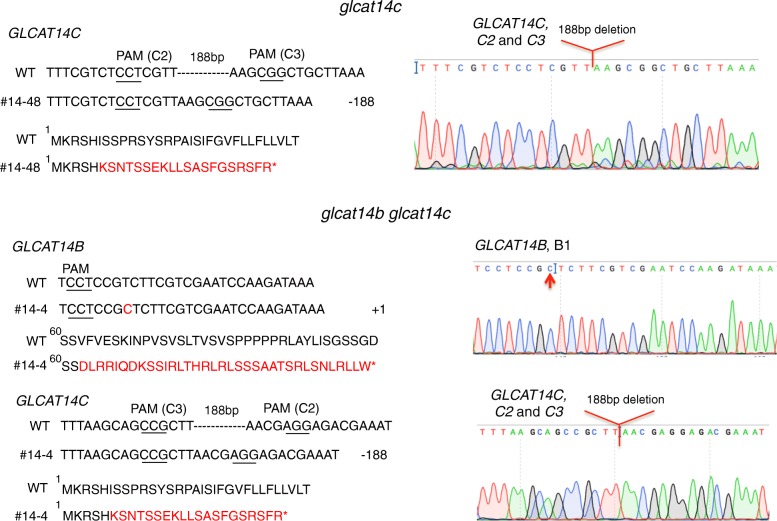
Sequencing results of the target sites for the *glcat14b glcat14c* double mutant (line #14–4). *GLCAT14B* contained a 1 bp insertion, which resulted in changes to the protein sequence and a pre-mature stop codon (red); *GLCAT14C* contained a 188 bp deletion between the C2 and C3 targeting sites as indicated in red on the chromatogram on the right

**Fig. 5:**
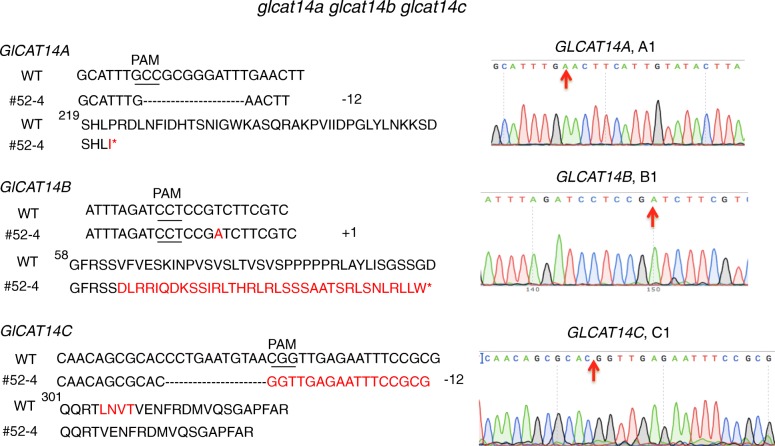
Sequencing results of three target sites (A1, B1, and C1) in the *glcat14a glcat14b glcat14c* triple mutant (line #52–4). *GLCAT14A* contained a 12 bp deletion, which resulted in pre-mature stop codon; *GLCAT14B* contained a 1 bp insertion, which resulted in a pre-mature stop codon in its protein sequence; *GLCAT14C* contained a 12 bp deletion, which resulted in the loss of four amino acids (red). Sequences of the mutation sites are indicated by red arrows on the chromatograms on the right

Three *glcat* homozygous mutants, namely *glcat14b*, *glcat14a glcat14b*, and *glcat14a glcat14b glcat14c* were generated in the T1 generation using the construct shown in Fig. 2B (1). The *glcat14b* mutant contained a 1 bp insertion at B1, while the *glcat14a glcat14b* mutant (line #17–5) contained 1 bp insertions at both A1 and B1 (Fig. 3). These mutations occurring in *glcat14b* and *glcat14a glcat14b* resulted in pre-mature stop codons for both genes. Cas9-free mutants were obtained for these two mutant lines in the T2 generation. Moreover, *glcat14c* and *glcat14b glcat14c* mutants were generated using the gene construct shown in Fig. 2B (2). Both mutant lines contained a 188 bp deletion between C2 and C3, while the *glcat14b glcat14c* double mutant line (line #14–4) additionally contained a 1 bp insertion at B1 (Fig. 4). Homozygous Cas9-free *glcat14c* and *glcat14b glcat14c* mutants were obtained in the T2 generation. Furthermore, one *glcat14a glcat14b glcat14c* triple mutant (line #52–4) was identified in the T2 generation using the construct in Fig. 2B (1). This mutant had a 12 bp deletion at A1, a 1 bp insertion at B2, and a 12 bp deletion at C1 (Fig. 5). In this triple mutant, gene editing events in gRNA A1 and B2 resulted in pre-mature stop codons, while the mutation at C1 caused the loss of four amino acids from its protein sequence (Fig. 5).

### Quantification of β-D-Gal-Yariv precipitated AGPs in the various *glcat* mutants

**Fig. 6:**
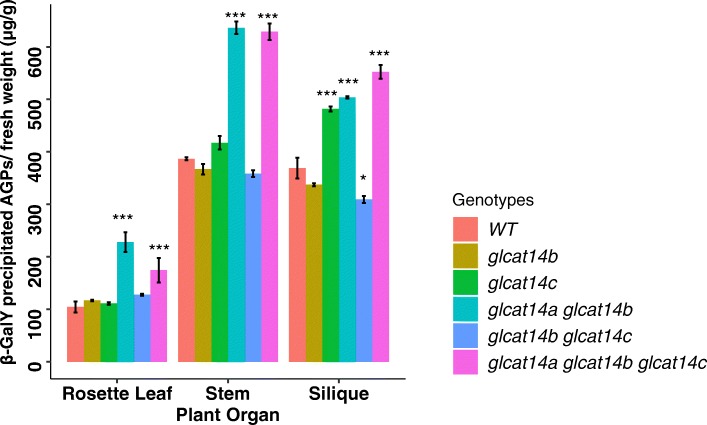
Quantification of AGP content of the various *glcat* mutants in different organs of 40-day-old plants. Single mutants did not show much change in their AGPs across different tissues except for *glcat14c* that contained more AGPs in the siliques. Both *glcat14a glcat14b* and *glcat14a glcat14b glcat14c* showed increases in AGP content in rosette leaves, stems, and siliques compared to other single mutants and WT across tissues, AGPs were measured as microgram per gram of fresh weight. Statistical differences were determined by two-way ANOVA, followed by the Tukey’s honestly significant difference test (* *P* < 0.05; ** *P* < 0.01; *** *P* < 0.001)

β-D-Gal-Yariv is a synthetic dye that is often used for AGPs purification as it can bind to the β-1,3-galactose backbone on AGPs; thus, it is an ideal reagent to facilitate characterization of the glycan structure of AGPs [22]. Here, β-D-Gal-Yariv was used to precipitate AGPs from rosette leaf, stem, and silique tissues of various *glcat* mutants (Fig. 6). The single mutants and the *glcat14b glcat14c* double mutant did not show much difference in the AGP content except for *glcat14c*, which showed an ~ 25% increase in AGP content only in silique but not in other organs. The *glcat14a glcat14b* double mutant and the *glcat14a glcat14b glcat14c* triple mutant, however, showed increases in AGP content in all three organs examined, namely rosette leaf, stem, and silique*.* Specifically, *glcat14a glcat14b* showed ~ 60%, ~ 50%, and ~ 25% increases in AGP content in rosettes, stems, and silique, respectively; whereas *glcat14a glcat14b glcat14c* showed ~ 50%, increases in AGP content in all three organs.

### Monosaccharide composition analyses

Previous monosaccharide composition analysis of *glcat14a* from 14-day-old seedlings found that there was a slight increase of Gal in 3-, 6- and 3,6-linkages in the AGPs extracted from *glcat14a* compared to that of WT, while the amount of GlcA in *glcat14a* was similar to that of WT [14]. Here, monosaccharide composition analyses were done by HPAEC-PAD for AGPs extracted from 40-day-old *glcat* mutants and WT. As seen in Table 1, single *glcat* mutants did not show notable differences in the amounts of most sugars, including GlcA, compared to WT with the exception of *glcat14c*, which showed an elevated amount of Gal and a reduced amount of GlcA. In contrast, the higher-order *glcat* mutants, namely *glcat14a glcat14b*, *glcat14b glcat14c*, and *glcat14a glcat14b glcat14c*, exhibited 27, 12, and 40% reductions in the amount of GlcA, respectively. Interestingly, these higher order mutants also displayed increased amounts of Ara and Gal compared to WT. Furthermore, the *glcat14a glcat14b glcat14c* mutant exhibited more pronounced reductions of GlcA than the double mutants; nonetheless, the GlcA content in the triple mutant was not completely eliminated.

**Table 1:** Monosaccharide composition analysis of AGPs extracted from aerial part of 40-day-old *glcat* CRISPR mutants and WT (Col-0)*

	Fuc	Rha	Ara	Gal	Glu	Xyl	Man	GalA	GlcA
**WT**	2.8 ± 0.7	4.8 ± 1.0	26.3 ± 2.4	39.1 ± 0.9	4.2 ± 0.3	7.0 ± 1.4	3.1 ± 0.6	5.0 ± 0.8	6.0 ± 1.3
***glcat14b***	2.7 ± 0.4	5.1 ± 0.6	24.6 ± 0.9	38.2 ± 0.4	3.7 ± 2.2	8.5 ± 1.2	3.3 ± 0.6	7.6 ± 1.4	6.1 ± 0.9
***glcat14c***	2.1 ± 0.4	4.4 ± 0.7	25.7 ± 2.1	41.5 ± 0.1	4.5 ± 0.2	6.7 ± 1.0	2.8 ± 0.4	7.2 ± 0.5	5.4 ± 0.9
***glcat14a glcat14b***	2.2 ± 0.2	3.4 ± 0.5	30.5 ± 3.8	42.3 ± 1.0	3.2 ± 1.0	5.6 ± 0.4	2.8 ± 0.2	4.5 ± 0.0	4.4 ± 0.4
***glcat14b glcat14c***	2.4 ± 0.2	4.1 ± 1.0	27.5 ± 0.4	44.7 ± 0.4	4.1 ± 0.1	7.0 ± 0.2	2.8 ± 0.0	3.8 ± 0.2	5.3 ± 0.4
***glcat14a glcat14b glcat14c***	2.8 ± 0.4	4.0 ± 1.2	30.7 ± 1.6	41.2 ± 1.0	3.6 ± 0.3	7.0 ± 0.7	2.7 ± 0.5	4.1 ± 1.3	3.6 ± 1.2

^*^Values are relative to total sugar composition (expressed as mol %) of triplicate assays ± SE

**Fig. 7:**
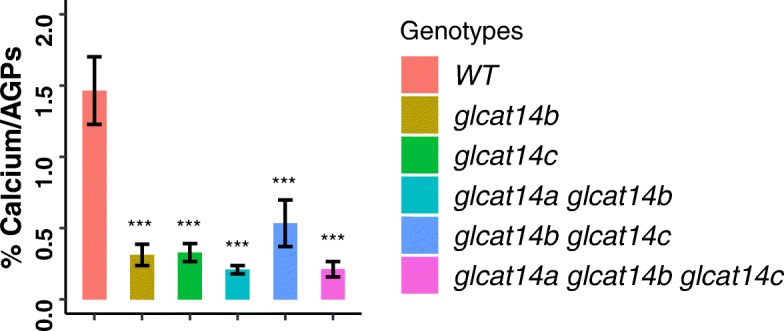
Quantification of calcium content of AGPs extracted from the aerial portion of 40-day-old plants. All the *glcat* mutants showed significant reductions of calcium binding. Statistical differences were determined by one-way ANOVA, followed by the Tukey’s honestly significant difference test (*** *P* < 0.001)

Previous research detected calcium associated with certain AGPs, including gum arabic and AGPs isolated from cell cultures of Arabidopsis, broccoli, carrot, tobacco, and tomato [23]. Lamport hypothesized that the negatively charged GlcA residues on AGPs may serve as sites for such calcium binding [24]. To examine whether calcium binding by AGPs is altered in our *glcat* mutants, we extracted AGPs from the aerial portion of Arabidopsis plants and measured the amount of associated calcium using a colormetric assay [25]. As shown in Fig. 7, WT Arabidopsis contained 1.5% Ca^2+^ per μg AGP, whereas all the *glcat* mutants contained 0.5% Ca^2+^ per μg AGP. Interestingly, the single *glcat* mutants had reductions in calcium binding comparable to the higher-order mutants. These results indicate that GlcA on AGPs is responsible for binding calcium and loss of any of the three GLCATs resulted in major loss of calcium binding of AGPs.

### *glcat14a glcat14b* and *glcat14a glcat14b glcat14c* exhibited delayed germination

**Fig. 8:**
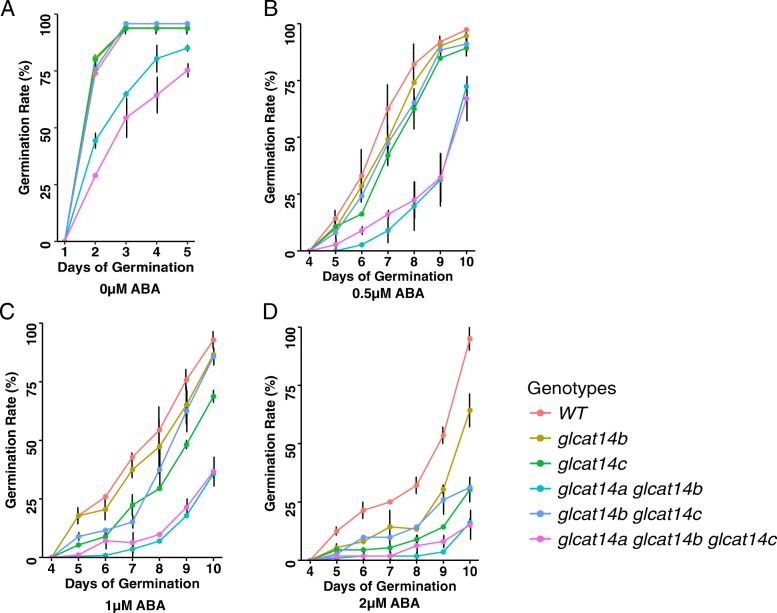
Germination percentages of the *glcat* mutants under 0, 0.5, 1, and 2 μM ABA. **a **Germination percentages of *glcat* mutants under normal ½ MS without ABA treatment. Higher-order *glcat* mutants showed delayed germination compared to *glcat* single mutants and wild-type (WT). Fifty seeds of each genotype were used for germination, with three replicates. **b**, **c**, and **d** Germination percentages of *glcat* mutants in the presence of 0.5, 1, and 2 μM ABA; germination of *glcat14a glcat14b* and *glcat14a glcat14b glcat14c* were inhibited under all three ABA concentrations, whereas other *glcat* mutants exhibited the most inhibition with 1 and 2 μM ABA

A significant delay in germination was observed in both post-harvested (> 3 months) and freshly-harvested seeds of *glcat14a glcat14b* and *glcat14a glcat14b glcat14c* seeds. Only ~ 40% of *glcat14a glcat14b* and ~ 30% of *glcat14a glcat14b glcat14c* seeds germinated by the 2nd day after sowing compared to ~ 75% seed germination for the other genotypes (Fig. 8a). By the 3rd day most of the WT, *glcat14b*, *glcat14c,* and *glcat14b glcat14c* seeds germinated, while only ~ 60% and ~ 55% of *glcat14a glcat14b* and *glcat14a glcat14b glcat14c* seeds respectively germinated. Overall, the germination time of N_50_ (50% germination rate) for most *glcat* mutants and WT was ~ 1.5 days compared to 2.5 days to 3 days for the *glcat14a glcat14b* and *glcat14a glcat14b glcat14c* mutants, respectively. This delayed germination phenotype for these *glcat* mutants, however, did not affect their ultimate ability to germinate, as most seeds eventually germinated (Fig. 8a).

Given that *GLCAT14A* and *GLCAT14B* are both highly expressed in the micropylar endosperm and the weakening of the endosperm cell wall is negatively regulated (i.e., delayed) by plant hormones such as ABA, we germinated *glcat* mutant seeds in the presence of 0.5, 1, and 2 μM ABA. The germination of WT seeds shared a similar trend to most *glcat* mutants; all of them germinated faster than *glcat14a glcat14b* and *glcat14a glcat14b glcat14c* in the presence of 0.5 μM ABA (Fig. 8b). In the presence of 1 μM ABA, *glcat14c* and *glcat14b glcat14c* germinated later than WT along with a more severe delay of germination observed for *glcat14a glcat14b* and *glcat14a glcat14b glcat14c* (Fig. 8c). In the presence of 2 μM ABA, all the *glcat* mutants demonstrated delayed germination compared to WT (Fig. 8d).

### *glcat14a glcat14b* and *glcat14a glcat14b glcat14c* are hypersensitive to stratification conditions

**Fig. 9:**
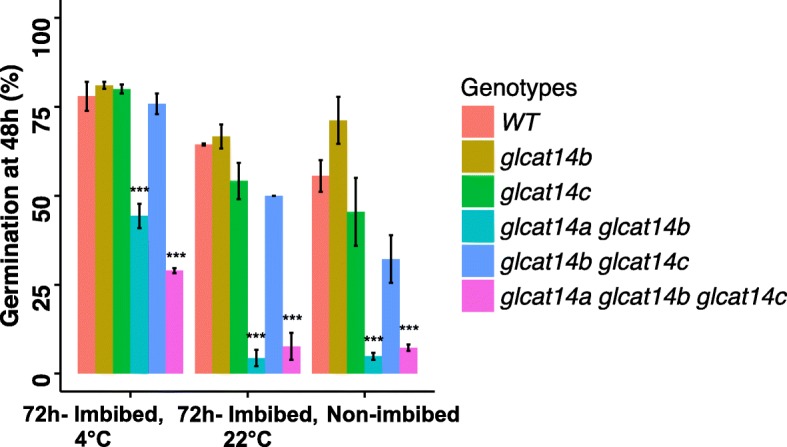
Germination percentages of the *glcat* mutants and WT under different stratification conditions. Seeds of the *glcat* mutants and WT were treated by normal stratification (water imbibition for 72 h, 4 °C), stratification at room temperature (water imbibition for 72 h, 22 °C), and no stratification (no imbibition) before sowing. Germination rates were measured 48 h after sowing

**Fig. 10:**
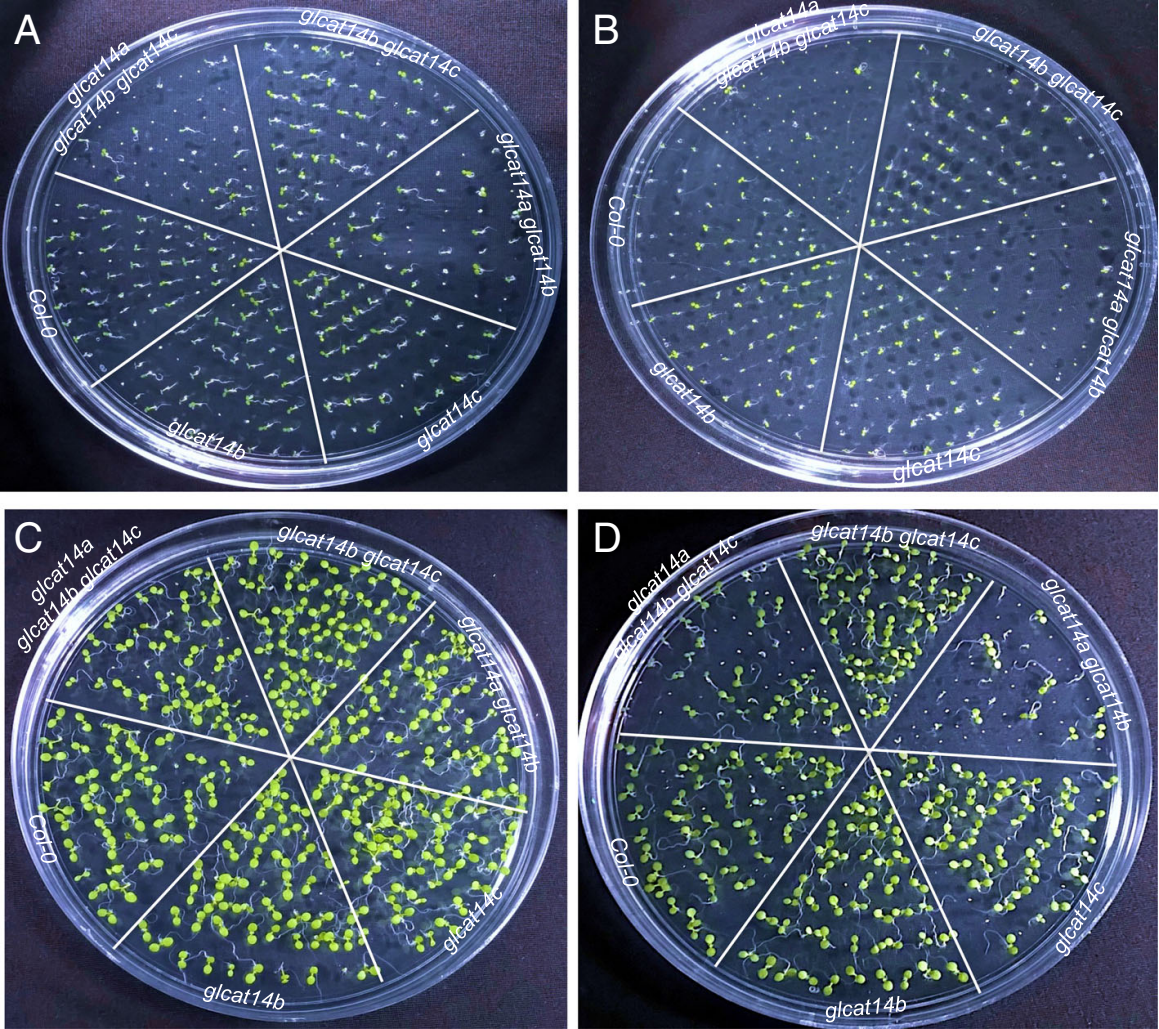
Germination assays of the *glcat* mutants. The *glcat* mutants and WT seeds were treated by normal stratification (**a** and **c**) and no stratification (**b** and **d**) before sowing; germination was examined 3 days (**a** and **b**) and 6 days (**c** and **d**) after sowing

Stratification at cold temperature is a common strategy to release dormancy of Arabidopsis seeds. Stratification can especially speed up the germination process of freshly harvested seeds, which contain a higher ABA content than post-harvested (> 3 months) seeds. To investigate whether the delayed germination phenotype observed for *glcat14a glcat14b* and *glcat14a glcat14b glcat14c* is related to stratification conditions, freshly harvested seeds from the *glcat* mutants and WT were treated with normal stratification (water imbibition for 72 h, 4 °C), stratification at room temperature (water imbibition for 72 h, 22 °C), and no stratification (no imbibition) before sowing. Germination rates were then measured 48 h after sowing. While stratification at room temperature and no stratification reduced germination percentages in all seeds compared to normal stratification, the *glcat14a glcat14b* and *glcat14a glcat14b glcat14c* seeds were especially sensitive to these two treatments and exhibited significant delays in germination (Figs. 9 and 10). These experiments indicate that the *GLCATs,* especially *GLCAT14A* and *GLCAT14B* together, play a role in the transition of dormant seeds to non-dormant seeds and is promoted by cold stratification.

### Higher-order *glcat* mutants showed less trichome branching, reduced root hair length, and reduced plant height

**Fig. 11:**
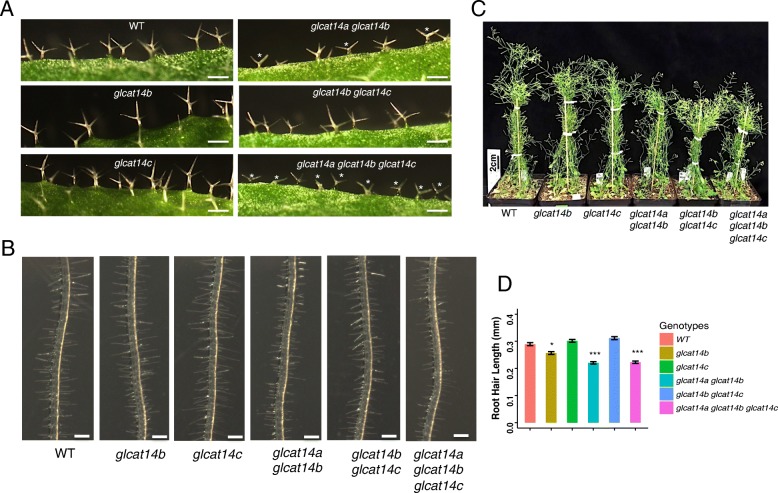
Phenotypic differences observed in the *glcat* mutants compared to WT. **a** Trichomes from rosettes of 30-day-old plants. Abnormal trichome branching is indicated by white asterisks. Scale bar = 300 μm. **b** Root hairs of 7-day-old seedlings. Scale bar = 1 mm. **c** Growth phenotype of 40-day-old *glcat* mutants and WT. **d** Shorter root hairs were found in *glcat14b*, *glcat14a glcat14b*, and *glcat14a glcat14b glcat14c.* (* *P* < 0.05; ** *P* < 0.01; *** *P* < 0.001)

Mutants that have both *GLCAT14A* and *GLCAT14B* knocked out also showed defects in trichome branching and reduction in root hair growth. Both *glcat14a glcat14b* and *glcat14a glcat14b glcat14c* had smaller trichomes and reduced trichome branching compared to WT, whereas single *glcat* mutants and *glcat14b glcat14c* phenocopied WT in trichome development (Fig. 11a). Moreover, root hair length was reduced in *glcat14b*, *glcat14a glcat14b*, and *glcat14a glcat14b glcat14c* compared to WT (Fig. 11b and d). Furthermore, the *glcat* double and triple mutants demonstrated reductions in plant height (Fig. 11c). The above mutant phenotypes indicate that GlcA residues on AGPs likely contribute to cell differentiation and tip-focused growth.

### *glcat14a glcat14b* and *glcat14a glcat14b glcat14c* contain defective pollen

**Fig. 12:**
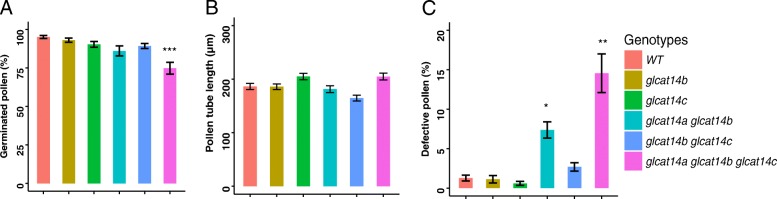
Pollen germination rate and pollen shape of the *glcat* mutants and WT. **a** Pollen germination percentages; only the *glcat14a glcat14b glcat14c* triple mutant showed a lower pollen germination percentage compared to WT. **b** Pollen tube lengths; all the *glcat* mutants exhibited similar pollen tube lengths compared to WT. **c** Defective pollen; the *glcat14a glcat14b* and *glcat14a glcat14b glcat14c* mutants contained significant amounts of defective pollen. All measurements were taken 3 h after incubation of pollen grains on pollen germination media. Approximately 200 pollen grains were measured for each genotype with three replicates. (* *P* < 0.05; ** *P* < 0.01; *** *P* < 0.001)

**Fig. 13:**
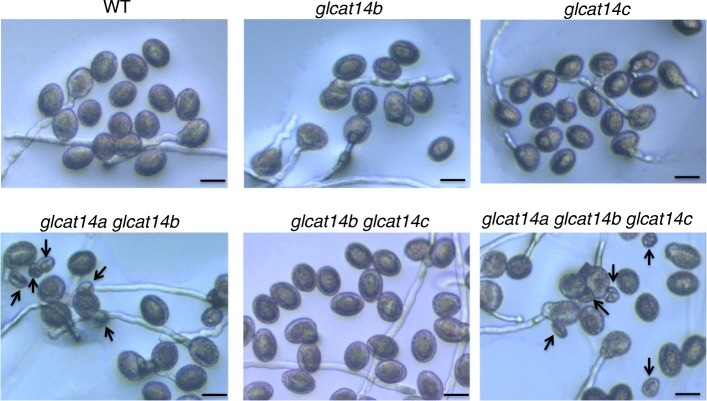
In vitro pollen germination of the *glcat* mutants and WT. Higher-order *glcat* mutants showed a significant number of defective pollen as indicated by black arrows. All pictures were taken 3 h after incubation of pollen grains on pollen germination media. Scale bar = 25 μm

In vitro pollen germination experiments were done to examine pollen phenotypes of the various *glcat* mutants. Only *glcat14a glcat14b glcat14c* exhibited a lower pollen germination rate compared to WT, whereas the pollen tube lengths of all *glcat* mutants were not significantly different from WT (Fig. 12a and b). In addition, *glcat14a glcat14b* and *glcat14a glcat14b glcat14c* mutants exhibited a significant number of defective pollen (Fig. 12c). The defective pollen was much smaller in size compared to normal pollen and failed to germinate (Fig. 13).

### Disruptions of the *GLCATs* resulted in shorter silique lengths and reduced seed set

**Fig. 14:**
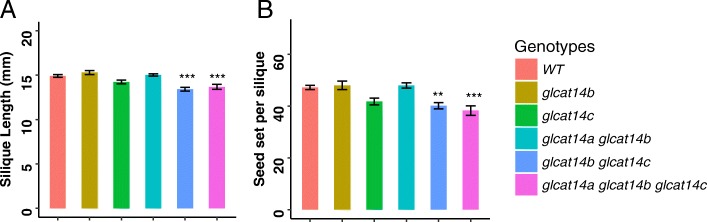
Silique length and seed set of the *glcat* mutants and WT. **a** Silique length; reductions in silique length were observed in *glcat14b glcat14c* and *glcat14a glcat14b glcat14c***b** Seed set; *glcat14b glcat14c* and *glcat14a glcat14b glcat14c* showed reduced seed set (* *P* < 0.05; ** *P* < 0.01; *** *P* < 0.001)

Only the *glcat14b glcat14c* and *glcat14a glcat14b glcat14c* mutants exhibited shorter silique lengths and reductions in seed set compared to WT (Fig. 14). *GLCAT14C* showed the highest expression in silique with *GLCAT14A* and *GLCAT14B* showing more modest expression based on our RT-qPCR data (Fig. 1).

### Higher-order *glcat* mutants had less adherent seed coat mucilage

**Fig. 15:**
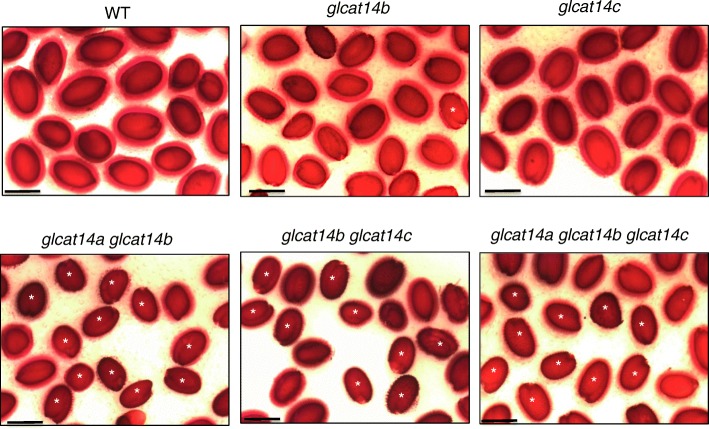
Ruthenium red staining of seeds from the *glcat* mutants and WT. All *glcat* higher-order mutants exhibited less seed mucilage content compared to *glcat* single mutants and WT. Seeds for each genotype were first imbibed in 1 mL water for 1 h with gentle shaking and subsequently incubated with 1 mL 0.01% ruthenium red for 0.5 h with gentle shaking. Seeds were ultimately rinsed with 1 mL water three times and observed by light microscopy, *N* = 30, Scale bar = 500 μm

Another notable phenotype of *glcat* mutants is the significant reduction in adherent seed coat mucilage. Seeds of *glcat14a glcat14b*, *glcat14b glcat14c*, and *glcat14a glcat14b glcat14c* showed dramatic reductions in adherent seed mucilage compared to WT as determined by staining with ruthenium red staining, which binds to acidic biopolymers such as pectin (Fig. 15).

## Discussion

### CRISPR-Cas9 approach to generate higher-order *GLCAT* mutants

Gene redundancy is common for genes encoding enzymes that function in cell wall biosynthesis including glycotransferases (GTs). In order to better understand the function of redundant gene families, higher order knockout mutants, mutants in which multiple genes in the family are inactivated, need to be generated and studied. Traditional genetic crossing/breeding approaches to obtain higher order mutants, however, are both time-consuming and complicated by genetic linkage. Compared to traditional breeding, CRISPR-Cas9 gene editing has been recently used to study redundant gene families in several plant species such as rice, wheat, cabbage, tomato and Arabidopsis [21, 26–29]. The research reported here, however, represents the first application of CRISPR/Cas9 to understand GTs families in Arabidopsis. Here, we show that CRISPR/Cas9 gene editing was successfully used to create both single *glcat* mutants as well as higher order *glcat* mutants. Without this approach, it would have been extremely difficult to generate *glcat14a glcat14b* and higher order mutants, as the two genes are approximately 12 cM apart on chromosome 5. As discussed below, the single *glcat* mutants (*glcat14b* and *glcat14c)* largely phenocopied WT and did not show changes in the monosaccharide composition on their AGPs, whereas disruption of two or more GLCATs had effects on both the AGP sugar composition as well as several significant developmental/physiological phenotypes in Arabidopsis.

Two multiplexed CRISPR constructs (Fig. 2B) were created to target the three known *GLCAT* gene family members, namely, *GLCAT14A*, *GLCAT14B*, and *GLCAT14C*. Following transformation with these constructs, a number of single, double, and triple *glcat* mutants were generated. Indel mutations created in *glcat14b*, *glcat14c*, *glcat14a glcat14b*, and *glcat14b glcat14c* all resulted in pre-mature stop codons (Figs. 3, 4). In the *glcat14a glcat14b glcat14c* triple mutant, mutations in *GLCAT14A* and *GLCAT14B* resulted in pre-mature stop codons, whereas mutation in *GLCAT14C* caused the loss of four amino acids in the GLCAT Pfam domain (Pf02485) (Fig. 5).

To evaluate potential off-target mutations caused by CRISPR-Cas9, CRISPR-P 2.0 software [30] was used to identify off-target candidates. Four sites located in the coding regions and showed off-target scores between 0.1 and 0.2 were identified (Additional file 1: Table S4). These sites were amplified and sequenced in the mutants. No off-target mutations were identified in any of our *glcat* mutants.

### CRISPR-induced *GLCAT* mutations alter AGP sugar composition and calcium binding

AGP monosaccharide composition analyses of mature plants showed that *glcat14a glcat14b*, *glcat14b glcat14c*, and *glcat14a glcat14b glcat14c* exhibited approximately 27, 12, and 40% reductions of GlcA, respectively (Table 1). In addition, *glcat14c*, *glcat14a glcat14b*, *glcat14b glcat14c*, and *glcat14a glcat14b glcat14c* exhibited more Gal (~ 5–12%) and more Ara (~ 15%) compared to WT. This is in general agreement with previous work on a *glcat14a* T-DNA insertion mutant, which detected 12% more Gal and 12% less Ara in AGPs isolated from young seedlings, although differences in GlcA levels were not detected by monosaccharide composition analysis this study [14]. Our findings are also consistent with the suggestion that GlcA residues on AGPs may serve to terminate further elongation of β-1,3- and β-1,6-galactan sugar additions to AGPs [14], such that reduced levels of GlcA lead to additional elongation and incorporation of Gal and Ara residues (Table 1).

Knocking out any of the *GLCAT* genes also led to significantly less calcium binding by AGPs compared to WT (Fig. 7), suggesting that GlcA, as the only negatively charged sugar on AGPs, plays a key role in calcium binding by AGPs. This provides the first direct experimental evidence specifically linking GlcA residues on AGPs with calcium binding, corroborating Lamport’s observation that AGPs bind calcium [23].

In order to examine whether the amount of glycosylated AGPs was altered in the various organs of the *glcat* mutants, we used β-Gal Yariv reagent to quantitate glycosylated AGPs from rosette leaves, stems, and siliques (Fig. 6). Both the *glcat14a glcat14b* double mutant and the *glcat14a glcat14b glcat14c* triple mutant exhibited ~ 30–60% increases of AGPs from rosettes, stems and siliques compared to WT. In addition, the *glcat14c* single mutant exhibited a ~ 25% increase of AGPs in the siliques. Such increases in AGP content are consistent with the idea that the loss of GlcA residues results in the elongation of β-1,3-galactose backbone and the addition of β-1,6-galactose side chains in AGPs, as revealed by β-Gal Yariv reagent and corroborated by monosaccharide analysis (Table 1). These results indicate that *GLCAT14A* and *GLCAT14B* play redundant roles in catalyzing the transfer of GlcA to AGPs in all three organs.

### *GLCAT14A* and *GLCAT14B* function redundantly in regulating seed germination

Among the *glcat* mutants, only *glcat14a glcat14b* and *glcat14a glcat14b glcat14c* exhibited 1–1.5 day delays in seed germination compared to WT (Figs. 8 and 9). Such delays in germination were exacerbated in seeds without normal (cold) stratification. This indicates that *GLCAT14A* and *GLCAT14B* function redundantly in regulating seed germination and such regulation most likely involves enhancing the degradation of the cell wall in the micropylar endosperm, where both genes are highly expressed. Thus, cold temperature imbibition likely loosens the micropylar endosperm cell wall to facilitate germination. A number of other cell wall enzymes were shown to be involved in seed germination such as expansin, β-1, 3-glucanase, endo-β-1,4 mannanase, pectin methylesterase, endotransglycosylase/hydrolases (XTH), α-xylosidase, and xylosyltransferases, all of which are also highly expressed and many of which are specifically expressed in the micropylar endosperm [31–36]. These enzymes likely play key roles in seed germination, as supported by expression analysis and by genetic mutant analysis. For example, knockout mutants of genes encoding endo-β-1,4 mannanase including *man5*, *man6*, and *man7* all exhibited a delayed germination phenotype in Arabidopsis. Moreover, a *man7* homolog also accumulated in the micropylar endosperm in *Lepidium sativum*, a close Arabidopsis relative [37].

In addition to the germination delays under normal conditions, all the *glcat* mutants also demonstrated even greater delays in the presence of 1–2 μM ABA. ABA inhibits only the 2nd phase of germination, which involves radical protrusion. The observed germination delay exhibited here by the *glcat* mutants in response to ABA indicates that GlcA residues on AGPs may function as a signaling molecules in such ABA-mediated germination.

### *GLCATs* involved in vegetative tip-focused growth in Arabidopsis

Among the *glcat* mutants, both *glcat14a glcat14b* and *glcat14a glcat14b glcat14c* displayed reductions in root hair length, trichome size, and trichome branching (Fig. 11). The role of AGPs in cell growth and expansion has long been shown by applying β-Gal-Yariv reagent to plant culture and observe growth inhibition [38]. β-Gal-Yariv, which specifically binds to the β, 1,3- galactose sugar backbone on AGPs, can inhibit root cell expansion and proliferation in vitro [39]. The *galt2 galt5* knockout mutant, affecting two GALTs involved in adding the first galactose residues to hydroxyproline residues on AGPs, also demonstrated reductions in root hair length [8]. Root hair and trichome formation are two examples of tip-focused growth driven by a cytoplasmic calcium gradient generated at the tip [40]. Recent studies found that AGPs can bind and release calcium in a pH-dependent fashion, presumably by their negatively charged GlcA residues [23]. Consequently, it was suggested that AGPs serve as extracellular calcium capacitors and are involved in calcium-mediated signaling, such that lower pH levels generated by actin mediated H^+^-ATPase disassociates calcium from extracellular AGPs and releases it into the cytosol, generating a cytoplasmic calcium influx [24]. This process may thus be important for various developmental processes, including root hair and trichome growth. A calcium-binding assay performed here for *glcat14a glcat14b* and *glcat14a glcat14b glcat14c* indeed found significant less calcium binding to AGPs compared to WT (Fig. 7). Therefore, the shorter root hair lengths and deformed trichome growth observed in our *glcat* mutants may be caused by reduced calcium influx in these tissues as brought about by the reduction of GlcA levels, and hence calcium levels, in the extracellular AGPs in these tissues.

There are other potential mechanisms of action for AGPs that could account for impaired root tip growth observed in the *glcat* mutants, as well as for the other mutant phenotypes reported in this study. For example, AGPs may control growth by interacting with other cell wall components. AGPs are often associated with pectin and in some cases covalently interact with them [41]. Studies on SOS5/FLA4, which is a chimeric AGP, found that *sos5* showed significant reduction in the seed mucilage content, and suggested that SOS5 interacts with and mediates pectin network assembly in seed mucilage [42]. In another study, AGPs were shown not only to covalently interact with pectin, but also to covalently interact with arabinoxylan (hemicellulose) [43]. More precisely, this study demonstrated that GlcA residues in AGPs were covalently linked to Rha residues in pectin [43]. Such structural interactions could thus be involved in controlling cell wall rigidity and extensibility. Another recent study on UPEX1, which is an AGP-specific glycosyltransferase in the GT31 family, also suggested that AGPs and xylan interact during exine cell wall deposition during pollen development [44]. Similar to the idea that un-methyl esterified pectin can form “egg-box” structures through Ca^2+^ binding that has been known to control cell wall integrity, the recently proposed AGP- Ca^2+^ model makes the negatively charged GlcA residues other potential Ca^2+^ binding sites on the cell surface to regulate cell wall integrity.

AGPs may also act as sensors and/or signaling molecules. The presence of AGPs at the plasma membrane/cell wall interface by virtue of the GPI anchoring domains present on many AGPs position them at an ideal location for sensing cell wall perturbations and transmitting information to associated signaling molecules such as receptor-like kinases. Physical interaction between AGPs and receptor like kinases (RLKs) is likely; this is based largely on genetic data. Specifically, *sos5/fla4* mutants and two Leu-rich repeat RLKs mutants, *fei1* and *fei2,* exhibited similar root swollen phenotypes as did *sos5 fei1 fei2* triple mutants, indicating nonadditive genetic interaction [45]. Thus, it was proposed that SOS5 is in the same genetic pathway as FEI1 and FEI2 and that SOS5 is a ligand for FEI1 and FEI2.

### *GLCATs* contribute to reproductive growth in Arabidopsis

Phenotypic analyses of the *glcat* mutants also indicates a role for the GLCATs in plant reproduction. Both *glcat14a glcat14b* and *glcat14a glcat14b glcat14c* displayed significant percentages of defective pollen that are much smaller than normal pollen and fail to germinate and *glcat14a glcat14b glcat14c* also exhibited a significantly lower pollen germination rate (Figs. 12 and 13). Furthermore, *glcat14b glcat14c* and *glcat14a glcat14b glcat14c* exhibited a reduction in silique length and seed set (Figs. 14). These observations relate to previous studies on AGPs. One of which found that *agp6* single mutants*, agp11* single mutants, and *agp6 agp11* double mutants display reduced pollen germination, pollen tube elongation, and pollen release [46]. The other study showed that AGP4, which is only expressed in the inflorescence, functions to prevent multiple pollen tubes from entering into one embryo sac in Arabidopsis, as *agp4* loss of function mutants failed to block multiple pollen tubes from entering the embryo sac [47]. Recent studies in *Torenia fournieri* identified a disaccharide sugar, β-methyl-glucuronosyl galactose (4-Me-GlcA-β-1,6-Gal) present on an AGPs secreted from the ovary that makes pollen tubes competent for ovule targeting/guidance [48]. Thus, GlcA residues on AGPs could play roles in ovular guidance and/or polytubey in Arabidopsis, but further research is needed to support these potential roles. As the only negatively charged sugar on AGPs, GlcA is likely responsible for calcium-binding by AGPs. Intracellular calcium signatures were detected at every step of the double fertilization process in Arabidopsis [49]. As such, GlcA residues on AGPs could function in fertilization via calcium signaling.

### *GLCATs* play an essential role in seed coat mucilage extrusion

Seed coat mucilage represents a useful model for studying genes involved in cell wall biosynthesis. As much as 90% of this mucilage is composed of pectin polysaccharides and less than 10% of the mucilage is made up of other cell wall component such as cellulose, galactoglucomannan, xyloglucan, xylan, and AGPs [50]. Disruptions of genes involved in cell wall biosynthesis often results in a reduction or loss of the seed mucilage [51]. Knocking out two of the AGP-specific GALTs, *galt2 galt5,* also resulted in a reduction of seed mucilage [8]. Mutations of other cell wall proteins such as *csla2*, *mum2*, and *fly1* also result in reductions of seed coat mucilage formation [52–54]. This study found that *glcat* double and triple mutants exhibited significant reductions in adherent seed mucilage as detected by ruthenium red staining (Fig. 15). There is insufficient information on role of GlcA in seed coat mucilage composition. In Arabidopsis, mucilage analyses either failed to detect or only detected less than 0.5 mol% GlcA [50, 51]. This study, for the first time, implicates that GlcA residues on AGPs play a substantial role in Arabidopsis seed coat mucilage formation as disruptions of two or more of the GLCATs resulted in a considerable loss of adherent seed mucilage. These GlcA residues, although minor components of the mucilage, may be important for covalent cross-linking with other cell wall components in the mucilage such as pectin, and specifically RG-1, as recently indicated by biochemical analysis of an extracellular AGP-pectin-arabinoxylan polymer, or alternatively, by ionic interactions with other negatively charged polymers (e.g., other AGPs or pectins) via calcium bridges [38, 40]. Reduction of GlcA on the AGPs may interfere with the normal structure of the primary cell wall thus impair the release or retention of the pectin-rich seed coat mucilage.

## Conclusions

The pioneering CRISPR-Cas9 approach used in this study has proven to be an efficient and effective way to characterize higher-order knockout mutants of the three membered *GLCAT* gene family. Our biochemical and phenotypic analyses indicate that *GLCAT14A* and *GLCAT14B* play a particularly redundant role in adding GlcA to AGPs in Arabidopsis. Nonetheless, mutant analyses showed that in fact all three *GLCATs* shared some degree of redundancy, as double and particularly triple mutants of *GLCATs* resulted in more severe phenotypic defects than single mutants. All the *GLCATs*, by virtue of their ability to add GlcA residues, confer the ability of AGPs to bind calcium. The concept that intracellular calcium originates by the influx of calcium ions from the plant cell wall/extracellular matrix is often overlooked in favor of the release from intracellular reservoirs (ER, vacuole) [55]. It is hypothesized that AGPs can serve as pH-regulated calcium capacitors and the source for intracellular calcium signals required for many plant growth and development processes [24]. Further studies will be required to examine potential calcium-related signaling pathways revealed by the *glcat* mutants. The observation that GlcA content was only reduced by ~ 50% on the AGPs in the *glcat14a glcat14b glcat14c* triple mutant indicates that additional GLCATs are likely involved in catalyzing the transfer of GlcA to AGPs. Thus, two important avenues of research to pursue in this area are: 1. Discovering which of the other members of the CAZy GT14 family may be responsible for adding GlcA residues to AGPs and 2. Understanding the exact mode or mechanism of action whereby GlcA residues and AGPs control specific developmental/physiological phenotypes, whether it be calcium-mediated signaling, altered cell wall structural interactions, and/or other AGP sensing/signaling scenarios.

## Methods

### Plant material

*Arabidopsis thaliana* (Columbia ecotype) was used as the WT, and it was originally obtained from the Arabidopsis Biological Research Center (ABRC), Columbus, Ohio, USA. We generated all the CRISPR mutants in this study using this *Col-0* background.

### In silico analysis of GLCAT14A, GLCAT14B, and GLCAT14C

Genomic DNA, protein sequences, and gene locations of *GLCAT14A*, *GLCAT14B*, and *GLCAT14C* were obtained from the TAIR website (https://www.arabidopsis.org/). Protein domain searches were done in Pfam (https://pfam.xfam.org/). Expression profiles of *GLCATs* based on RNA-seq data were obtained from the TRAVA database [18]. The transcriptomic data of seed development was obtained from the eFP browser (https://bar.utoronto.ca/efp/cgi-bin/efpWeb.cgi) [56].

### Guide RNA design and vector construction

All gRNAs were selected using the online software CRISPR-P 2.0 (http://crispr.hzau.edu.cn/CRISPR2/). Several rules regarding sequence and structural requirements for choosing a gRNA were as described [57]. First, the gRNA should have a high on-target and low off-target score based on the algorithm of CRISPR-P 2.0. Also, the gRNA should contain no less than 40% GC content for strong binding to the template DNA and have a G or C at the position 20 before the PAM sequence. The vector construction strategy was adopted from a published method [21]. Three template plasmids (pCBC-DT1, pCBC-DT2DT3, and pCBC-DT3DT4) and a binary vector (pHEE401E) were gifts from Dr. Qi-Jun Chen’s lab (Addgene plasmid #50590; #50591; #50592; #71287). Each CRISPR/Cas9 multiplexing construct, consisting of an individual gRNA cassette, which includes a gRNA sequence, a gRNA scaffold, an AtU6 promoter, and an AtU6 terminator, was first amplified from a specific template plasmid; see Additional file 1: Tables S1-S3 for specific gRNA and primer sequences. A detailed procedure for primer design and PCR reaction steps were described previously [21]. For the CRISPR/Cas9 construct containing three gRNAs: A1, B1, and C1, the gRNA A1 cassette was amplified using primers DT1-A1_F, DT1-A1_F0, and DT0-R2; while the B1 and C1 cassettes were amplified using primers DT2-B1_F, DT2-B1_F0, DT3-C1_R0, and DT3_C1_R. After amplifying each gRNA fragment, they were cloned into BsaI site of the pHEE401E binary vector by the Golden Gate cloning method [58]. For the CRISPR/Cas9 construct containing four gRNAs: A2, B1, C2, and C3, the gRNA C3 cassette was amplified using primers DT1-C3_F, DT1-C3_F0, and DT0_R2; the gRNA A2 cassette was amplified using primers DT2-A2_F2, DT2-A2_F0, and DT0-BsR3; the gRNA B1 and C2 cassettes were amplified using primers DT3-B1-BsF3, DT3-B1-F0, DT4-C2-R0, and DT4-C2-BsR. The four gRNAs fragments were then cloned into the BsaI site of the pHEE401E binary vector by the Golden Gate cloning method. The pHEE401E binary vector contains a maize codon optimized *Cas9* gene driven by egg cell-specific promoters (E.C 1.1 and E. C 1.2) [20]. The final constructs were transformed into *Agrobacterium* strain GV3101, which was then used for transforming Arabidopsis *Col-0* ecotype plants via the floral dip method [59]. A PCR-based method was used for initially detecting indel mutations, which were subsequently confirmed by Sanger sequencing [60].

### Quantitative RT-PCR

Total RNA was extracted from various tissues (imbibed seeds, germinated seeds, roots, rosettes, stems, open flowers, siliques) of *Col-0* Arabidopsis using Trizol (Life Technologies, Grand Island, NY, USA). RNA (1 μg) was used for first-strand cDNA synthesis along with an oligo (dT20) primer and SuperScript III reverse transcriptase (Thermo Scientific). The qPCR was performed using PerfeCTa SYBR Green SuperMix (Quanta Biosciences). A 20 μl reaction mixture was set up which contained 10 μl PerfeCTa SYBR Green SuperMix, 4 μl 10x diluted cDNA, and 0.6 μl forward/reverse primer (10 μM). The qPCR was run on an AriaMx Real-time PCR machine (Ohio University Genomics Facility: http://www.dna.ohio.edu/). PCR conditions were according to the PerfeCTa SYBR Green SuperMix protocol. Expression levels were calculated relative to the Arabidopsis *ACTIN2* gene. The qPCR primers are listed in Additional file 1: Table S5.

### AGPs quantification by β-D-gal-Yariv

AGPs were extracted from 40-day-old *glcat* mutants and WT using the β-D-Gal-Yariv precipitation method described by Lamport [61]. Briefly, tissues were harvested from rosette leaves, stems, and siliques of 40-day-old *glcat* and WT plants and pulverized using a mortar and pestle in the presence on liquid nitrogen. Approximately 0.3 g of pulverized tissues were mixed with 1 mL 2% CaCl_2_ and shaken at 150 rpm for 2 h. The ground tissues were then separated from the supernatant by centrifugation at 13,000 *g* for 10 min. Around 500 μL of supernatant was transferred to a new 1.5 mL centrifuge tube and mixed with 200 μL β-D-Gal-Yariv dissolved in 2% CaCl_2_ (1 mg/mL). Meanwhile, 500 μL 2% CaCl_2_ was mixed with 200 μL β-D-Gal-Yariv as control. After 2 h of β-D-Gal-Yariv precipitation, the pellet was obtained by centrifugation at 13,000 *g* for 10 min and then dissolved in 20 mM NaOH. The dissolved AGPs were quantified by measuring absorbance at OD_420_. Different concentrations of gum arabic (G9752, Sigma-Aldrich, St. Louis, MO, USA) dissolved in 1% CaCl_2_ were used to make the standard curve. Measurements for each genotype were done in triplicate.

### Monosaccharide composition analysis by high performance anion exchange chromatography with pulsed amperometric detection (HPAEC-PAD)

AGPs were extracted from the aerial part of 40-day-old *glcat* mutants and WT using the protocol developed by Lamport [61]. Pulverized plant tissue was mixed with 2% NaCl in 1:4 (w/v) ratio and shaken at 200 rpm for 3 h followed by centrifugation at 13,000 *g* for 30 min. Then 2 mL of 1 mg/mL β-D-Gal-Yariv reagent was mixed with the supernatant and allowed it to precipitate overnight. The next day, the precipitated AGPs were collected by centrifugation at 2000 *g* for 10 min, washed with 2% NaCl once, then resuspended in 2 mL H_2_O. The β-D-Gal-Yariv reagent was dissociated from the AGPs by adding ~ 25 mg sodium dithionite to each 2 mL AGPs sample and incubated at 50 °C for 15 min until the solution became yellow. A PD-10 column (GE Healthcare) was used for desalting, and the eluate was freeze-dried. Approximately 100 μg of AGPs dissolved in Milli-Q water were used for monosaccharide composition analysis. AGPs were hydrolyzed using 300 μl 2 N TFA at 121 °C for 90 min followed by removal of TFA using N_2_ gas. The sample was washed with isopropanol three times before dissolving it in 500 μL Milli-Q water. A standard sugar mixture (fucose, rhamnose, arabinose, galactose, glucose, xylose, mannose, galacturonic acid, and glucoronic acid) of various concentrations (5 nM, 2.5 nM, and 1.25 nM) were used for making the standard curve. Monosaccharide compositions were calculated as molar percentages (mol %). All samples and standards were subjected to high-performance anion-exchange chromatography with pulsed amperometric detection (HPAE-PAD) on a Dionex PA-20 (Thermo Fisher Scientific, Sunnyvale, CA, USA) essentially as described by [62].

### Calcium binding assay

AGPs were extracted from the aerial part of 40-day-old *glcat* mutants and WT plants as described above and dissolved in water. Approximately 1–2 mg/mL AGPs were dissolved in ultrapure water and the concentration was determined by β-D-Gal-Yariv precipitation method at OD_420_. A commercial calcium colormetric assay kit (MAK022, Sigma-Aldrich, St. Louis, MO, USA) was used for calcium measurement following the manufacturer’s protocol. In this assay, calcium ions from the AGP extracts form a complex with the o-cresolphthalein in the assay kit and cause a color change from transparent to pink. The amount of calcium was determined using a UV spectrometer at OD_575_ and a standard curve made with different concentrations of CaCl_2_.

### Germination experiment

Both freshly harvested and two-month post-harvested seeds of *glcat* and wild-type plants were used for germination experiments. Sterilized seeds were treated three ways: without stratification, stratification at 4 °C for 3 d, and 22 °C for 3 d in the dark. Seeds were sowed onto ½ MS and 1% sucrose agar plates. Germination percentages were counted from 1 to 5 d after sowing. Approximately 50 seeds were sown for each genotype with three replicates. For germination under ABA treatment, two-month post-harvested seeds were sterilized and stratified at 4 °C for 3 d. Seeds were sown onto ½ MS and 1% sucrose agar plates containing 0–2 μM ABA. Germination percentages were counted from 4 to 10 d after sowing. Approximately 50 seeds were sown for each genotype with three replicates.

### Root hair and trichome morphology

Seeds from *glcat* mutants and WT were sterilized and kept at 4 °C in the dark for 3 d for stratification before being sown onto ½ MS and 1% sucrose agar plates. Four-day-old seedlings were transferred onto ½ MS agar plates and kept in a growth chamber at 22 °C, 16 h light/ 20 °C, 8 h dark photoperiod. Root hairs were measured 5 mm from the root tip, 4 days after transfer. Approximately 300 root hairs were measured for each genotype with three replicates. For trichome morphology, trichomes from rosettes of 40-day-old *glcat* mutants and WT were used for imaging at 20x magnification with a Nikon SMZ1500 stereomicroscope.

### Evaluation of silique length and seed set

Siliques were collected from 40-day-old *glcat* mutants and WT for measurement of silique lengths and seed set. Seed set was measured after clearing the silique color with 70% ethanol overnight. Ten plants for each genotype were used for measurement with three replicates.

### In vitro pollen germination assay

Flowers from 35-day-old *glcat* mutants and WT were used for an in vitro pollen germination assay. The pollen germination medium contained 10% sucrose, 0.01% boric acid, 1 mM CaCl_2_, 1 mM Ca (NO_3_) _2_, 1 mM KCl, 0.03% casein enzymatic hydrolysate, 0.01% myo-inositol, 0.1 mM spermidine, 10 mM GABA, 500 μM methyl jasmonate, and 1% low-melting agarose. Pollen was taken from five flowers for each genotype and was incubated on pollen germination medium. Pollen shape, pollen germination rate, and pollen tube length were measured 3 h after incubation with a Nikon phot-lab2 microscope at 50x magnification. Around 300 pollen and pollen tubes were measured in an individual experiment with three replicates.

### Ruthenium red staining

Seeds of *glcat* mutants and WT were hydrated with 1 mL water and shaken at 200 rpm for 1 h to remove non-adherent mucilage. The water was removed with a pipet and replaced with 1 mL of 0.01% ruthenium red followed by shaking at 200 rpm for 30 min. Following removal of the stain with a pipet, seeds were rinsed with 1 mL water three times before being observed at 20x magnification under a Nikon SMZ1500 stereomicroscope.

## Supplementary information

**Additional file 1: **Five supplemental tables, three supplemental figures and their corresponding legends. **Table S1.** List of guide RNA sequences and their target genes. **Table S2.** List of primers used to assemble three gRNAs (A1, B1, and C1) into pHEE401E binary vector. **Table S3.** List of primers used to assemble four gRNAs (A2, B1, C2, and C3) into pHEE401E binary vector. **Table S4.** List of primers for sequencing off-targets. **Table S5.** List of primers used for qRT-PCR. **Figure S1.** Amino acid sequence alignment of GLCAT14A, GLCAT14B, and GLCAT14C. **Figure S2.** Expression profiles of *GLCAT14A*, *GLCAT14B*, and *GLCAT14C* obtained from the Klepikova Arabidopsis Atlas eFP Browser of the Bio-Analytic Resource for Plant Biology (BAR) [18]. The link for the expression profile of *GLCAT14A* can be found at http://bar.utoronto.ca/efp/cgi-bin/efpWeb.cgi?primaryGene=AT5G39990&dataSource=Klepikova_Atlas&modeInput=Absolute; The link for the expression profile of *GLCAT14B* can be found at http://bar.utoronto.ca/efp/cgi-bin/efpWeb.cgi?primaryGene=AT5G15050&dataSource=Klepikova_Atlas&modeInput=Absolute; The link for the expression profile of *GLCAT14C* can be found at http://bar.utoronto.ca/efp/cgi-bin/efpWeb.cgi?primaryGene=AT2G37585&dataSource=Klepikova_Atlas&modeInput=Absolute. **Figure S3.** Expression patterns of *GLCAT14A*, *GLCAT14B*, and *GLCAT14C* during seed development obtained from the Arabidopsis eFP Browser of the Bio-Analytic Resource for Plant Biology (BAR) (bar.utoronto.ca). The link for the expression profile of *GLCAT14A* during seed development can be found at http://bar.utoronto.ca/efp/cgi-bin/efpWeb.cgi?dataSource=Seed&modeInput=Absolute&primaryGene=At5g39990&secondaryGene=At3g27340&override=None&threshold=219.05&modeMask_low=None&modeMask_stddev=None; The link for the expression profile of *GLCAT14B* during seed development can be found at http://bar.utoronto.ca/efp/cgi-bin/efpWeb.cgi?dataSource=Seed&modeInput=Absolute&primaryGene=AT5G15050&secondaryGene=At3g27340&override=&threshold=17.75&modeMask_low=None&modeMask_stddev=None&gene_alias1=GlcAT14B&gene_alias2=; The link for the expression profile of *GLCAT14C* during seed development can be found at http://bar.utoronto.ca/efp/cgi-bin/efpWeb.cgi?dataSource=Seed&modeInput=Absolute&primaryGene=AT2G37585&secondaryGene=At3g27340&override=&threshold=18.13&modeMask_low=None&modeMask_stddev=None&gene_alias1=GlcAT14C&gene_alias2=.

## Data Availability

The datasets used and/or analyzed during the current study are available from the corresponding author upon reasonable request.

## Abbreviations

GLCAT: β-glucuronosyltransferase
AGP: Arabinogalactan protein
GlcA: Glucuronic acid
Hyp: Hydroxyproline
CAZy: Carbohydrate Active Enzyme database
GT: Glycosyltransferase
Guide RNA: gRNA

## References

[1] Ellis M, Egelund J, Schultz CJ, Bacic A (2010). Arabinogalactan-proteins: key regulators at the cell surface?. Plant Physiol.

[2] Showalter AM (2001). Arabinogalactan-proteins: structure, expression and function. Cell Mol Life Sci.

[3] Showalter AM, Basu D (2016). Glycosylation of arabinogalactan-proteins essential for development in Arabidopsis. Commun Integr Biol.

[4] Showalter AM, Basu D. Extensin and arabinogalactan-protein biosynthesis: glycosyltransferases, research challenges, and biosensors. Front Plant Sci. 2016;7. 10.3389/fpls.2016.00814. 10.3389/fpls.2016.00814 PMC4908140 27379116

[5] Liang Y, Faik A, Kieliszewski M, Tan L, Xu W-L, Showalter AM (2010). Identification and characterization of in vitro galactosyltransferase activities involved in arabinogalactan-protein glycosylation in tobacco and Arabidopsis. Plant Physiol.

[6] Basu D, Liang Y, Liu X, Himmeldirk K, Faik A, Kieliszewski M (2013). Functional identification of a hydroxyproline-O-galactosyltransferase specific for arabinogalactan protein biosynthesis in Arabidopsis. J Biol Chem.

[7] Ogawa-Ohnishi M, Matsubayashi Y (2015). Identification of three potent hydroxyproline O-galactosyltransferases in Arabidopsis. Plant J.

[8] Basu D, Tian L, Wang W, Bobbs S, Herock H, Travers A (2015). A small multigene hydroxyproline-O-galactosyltransferase family functions in arabinogalactan-protein glycosylation, growth and development in Arabidopsis. BMC Plant Biol.

[9] Suzuki T, Narciso JO, Zeng W, van de Meene A, Yasutomi M, Takemura S (2017). KNS4/UPEX1: a type II arabinogalactan β-(1,3)-galactosyltransferase required for pollen exine development. Plant Physiol.

[10] Dilokpimol A, Poulsen CP, Vereb G, Kaneko S, Schulz A, Geshi N (2014). Galactosyltransferases from Arabidopsis thaliana in the biosynthesis of type II arabinogalactan: molecular interaction enhances enzyme activity. BMC Plant Biol.

[11] Gille S, Sharma V, Baidoo EEK, Keasling JD, Scheller HV, Pauly M (2013). Arabinosylation of a Yariv-precipitable cell wall polymer impacts plant growth as exemplified by the Arabidopsis glycosyltransferase mutant ray1. Mol Plant.

[12] Wu Y, Williams M, Bernard S, Driouich A, Showalter AM, Faik A (2010). Functional identification of two nonredundant Arabidopsis α-(1→2)-fucosyltransferases specific to arabinogalactan proteins. J Biol Chem.

[13] Tryfona T, Theys TE, Wagner T, Stott K, Keegstra K, Dupree P (2014). Characterisation of FUT4 and FUT6 α-(1→2)-fucosyltransferases reveals that absence of root arabinogalactan fucosylation increases Arabidopsis root growth salt sensitivity. PLoS One.

[14] Knoch E, Dilokpimol A, Tryfona T, Poulsen CP, Xiong G, Harholt J (2013). A β–glucuronosyltransferase from Arabidopsis thaliana involved in biosynthesis of type II arabinogalactan has a role in cell elongation during seedling growth. Plant J.

[15] Dilokpimol A, Geshi N (2014). Arabidopsis thaliana glucuronosyltransferase in family GT14. Plant Signal Behav.

[16] Ye C-Y, Li T, Tuskan GA, Tschaplinski TJ, Yang X (2011). Comparative analysis of GT14/GT14-like gene family in Arabidopsis, Oryza, Populus, Sorghum and Vitis. Plant Sci.

[17] Mutwil M, Obro J, Willats WGT, Persson S (2008). GeneCAT--novel webtools that combine BLAST and co-expression analyses. Nucleic Acids Res.

[18] Klepikova AV, Kasianov AS, Gerasimov ES, Logacheva MD, Penin AA (2016). A high resolution map of the Arabidopsis thaliana developmental transcriptome based on RNA-seq profiling. Plant J.

[19] Le BH, Cheng C, Bui AQ, Wagmaister JA, Henry KF, Pelletier J (2010). Global analysis of gene activity during Arabidopsis seed development and identification of seed-specific transcription factors. Proc Natl Acad Sci U S A.

[20] Sprunck S, Rademacher S, Vogler F, Gheyselinck J, Grossniklaus U, Dresselhaus T (2012). Egg cell–secreted EC1 triggers sperm cell activation during double fertilization. Science..

[21] Wang Z-P, Xing H-L, Dong L, Zhang H-Y, Han C-Y, Wang X-C (2015). Egg cell-specific promoter-controlled CRISPR/Cas9 efficiently generates homozygous mutants for multiple target genes in Arabidopsis in a single generation. Genome Biol.

[22] Kitazawa K, Tryfona T, Yoshimi Y, Hayashi Y, Kawauchi S, Antonov L (2013). β-Galactosyl Yariv reagent binds to the β-1,3-galactan of arabinogalactan proteins. Plant Physiol.

[23] Lamport DTA, Várnai P (2013). Periplasmic arabinogalactan glycoproteins act as a calcium capacitor that regulates plant growth and development. New Phytol.

[24] Lamport DTA, Varnai P, Seal CE (2014). Back to the future with the AGP–Ca^2+^ flux capacitor. Ann Bot.

[25] Corns CM, Ludman CJ (1987). Some observations on the nature of the calcium-cresolphthalein complexone reaction and its relevance to the clinical laboratory. Ann Clin Biochem.

[26] Ma C, Liu M, Li Q, Si J, Ren X, Song H (2019). Efficient BoPDS gene editing in cabbage by the CRISPR/Cas9 system. Hortic Plant J.

[27] Wang W, Akhunova A, Chao S, Akhunov E. Optimizing multiplex CRISPR/Cas9-based genome editing for wheat. bioRxiv. 2016:051342. 10.1101/051342.

[28] Ma C, Zhu C, Zheng M, Liu M, Zhang D, Liu B (2019). CRISPR/Cas9-mediated multiple gene editing in Brassica oleracea var. capitata using the endogenous tRNA-processing system. Hortic Res.

[29] Wang D, Samsulrizal NH, Yan C, Allcock NS, Craigon J, Blanco-Ulate B (2019). Characterization of CRISPR mutants targeting genes modulating pectin degradation in ripening tomato. Plant Physiol.

[30] Liu H, Ding Y, Zhou Y, Jin W, Xie K, Chen L-L (2017). CRISPR-P 2.0: an improved CRISPR-Cas9 tool for genome editing in plants. Mol Plant.

[31] Chen F, Bradford KJ (2000). Expression of an expansin is associated with endosperm weakening during tomato seed germination. Plant Physiol.

[32] Cavalier DM, Keegstra K (2006). Two xyloglucan xylosyltransferases catalyze the addition of multiple xylosyl residues to cellohexaose. J Biol Chem.

[33] Leubner-Metzger G, Frundt C, Vogeli-Lange R, Meins F (1995). Class I [beta]-1,3-glucanases in the endosperm of tobacco during germination. Plant Physiol.

[34] Iglesias-Fernández R, Rodríguez-Gacio MC, Barrero-Sicilia C, Carbonero P, Matilla A (2011). Three endo-ß-mannanase genes expressed in the micropylar endosperm and in the radicle influence germination of Arabidopsis thaliana seeds. Planta..

[35] Müller K, Levesque-Tremblay G, Bartels S, Weitbrecht K, Wormit A, Usadel B (2013). Demethylesterification of cell wall pectins in arabidopsis plays a role in seed germination. Plant Physiol.

[36] Shigeyama T, Watanabe A, Tokuchi K, Toh S, Sakurai N, Shibuya N (2016). α-xylosidase plays essential roles in xyloglucan remodelling, maintenance of cell wall integrity, and seed germination in Arabidopsis thaliana. J Exp Bot.

[37] Morris K, Linkies A, Müller K, Oracz K, Wang X, Lynn JR (2011). Regulation of seed germination in the close arabidopsis relative Lepidium sativum: a global tissue-specific transcript analysis. Plant Physiol.

[38] Gao M, Showalter AM (1999). Yariv reagent treatment induces programmed cell death in Arabidopsis cell cultures and implicates arabinogalactan protein involvement. Plant J Cell Mol Biol.

[39] Willats WG, Knox JP (1996). A role for arabinogalactan-proteins in plant cell expansion: evidence from studies on the interaction of beta-glucosyl Yariv reagent with seedlings of Arabidopsis thaliana. Plant J Cell Mol Biol.

[40] Véry A-A, Davies JM (2000). Hyperpolarization-activated calcium channels at the tip of Arabidopsis root hairs. Proc Natl Acad Sci U S A.

[41] Immerzeel P, Eppink MM, de Vries SC, Schols HA, Voragen AGJ (2006). Carrot arabinogalactan proteins are interlinked with pectins. Physiol Plant.

[42] Griffiths JS, Tsai AY-L, Xue H, Voiniciuc C, Šola K, Seifert G, et al. SOS5 mediates Arabidopsis seed coat mucilage adherence and organization through pectins. Plant Physiol. 2014;165:991–1004. 10.1104/pp.114.239400 PMC4081351 24808103

[43] Tan L, Eberhard S, Pattathil S, Warder C, Glushka J, Yuan C (2013). An Arabidopsis cell wall proteoglycan consists of pectin and arabinoxylan covalently linked to an arabinogalactan protein. Plant Cell.

[44] Li WL, Liu Y, Douglas CJ (2017). Role of glycosyltransferases in pollen wall primexine formation and exine patterning. Plant Physiol.

[45] Xu S-L, Rahman A, Baskin TI, Kieber JJ (2008). Two leucine-rich repeat receptor kinases mediate signaling, linking cell wall biosynthesis and ACC synthase in Arabidopsis. Plant Cell.

[46] Coimbra S, Costa M, Jones B, Mendes MA, Pereira LG (2009). Pollen grain development is compromised in Arabidopsis agp6 agp11 null mutants. J Exp Bot.

[47] Pereira AM, Nobre MS, Pinto SC, Lopes AL, Costa ML, Masiero S (2016). “Love is strong, and you’re so sweet”: JAGGER is essential for persistent synergid degeneration and polytubey block in Arabidopsis thaliana. Mol Plant.

[48] Mizukami AG, Inatsugi R, Jiao J, Kotake T, Kuwata K, Ootani K (2016). The AMOR arabinogalactan sugar chain induces pollen-tube competency to respond to ovular guidance. Curr Biol CB.

[49] Denninger P, Bleckmann A, Lausser A, Vogler F, Ott T, Ehrhardt DW (2014). Male–female communication triggers calcium signatures during fertilization in *Arabidopsis*. Nat Commun.

[50] Western TL (2012). The sticky tale of seed coat mucilages: production, genetics, and role in seed germination and dispersal. Seed Sci Res.

[51] Voiniciuc C, Yang B, Schmidt MH-W, Günl M, Usadel B (2015). Starting to gel: how Arabidopsis seed coat epidermal cells produce specialized secondary cell walls. Int J Mol Sci.

[52] Yu L, Shi D, Li J, Kong Y, Yu Y, Chai G (2014). CELLULOSE SYNTHASE-LIKE A2, a glucomannan synthase, is involved in maintaining adherent mucilage structure in Arabidopsis seed. Plant Physiol.

[53] Dean GH, Zheng H, Tewari J, Huang J, Young DS, Hwang YT (2007). The Arabidopsis MUM2 gene encodes a beta-galactosidase required for the production of seed coat mucilage with correct hydration properties. Plant Cell.

[54] Voiniciuc C, Dean GH, Griffiths JS, Kirchsteiger K, Hwang YT, Gillett A (2013). Flying saucer1 is a transmembrane RING E3 ubiquitin ligase that regulates the degree of pectin methylesterification in Arabidopsis seed mucilage. Plant Cell.

[55] Hepler PK, Kunkel JG, Rounds CM, Winship LJ (2012). Calcium entry into pollen tubes. Trends Plant Sci.

[56] Winter D, Vinegar B, Nahal H, Ammar R, Wilson GV, Provart NJ (2007). An “electronic fluorescent pictograph” browser for exploring and analyzing large-scale biological data sets. PLoS One.

[57] Gagnon JA, Valen E, Thyme SB, Huang P, Ahkmetova L, Pauli A (2014). Efficient mutagenesis by Cas9 protein-mediated oligonucleotide insertion and large-scale assessment of single-guide RNAs. PLoS One.

[58] Engler C, Marillonnet S (2014). Golden Gate cloning. Methods Mol Biol.

[59] Clough SJ, Bent AF (1998). Floral dip: a simplified method for agrobacterium-mediated transformation of Arabidopsis thaliana. Plant J Cell Mol Biol.

[60] Hua Y, Wang C, Huang J, Wang K (2017). A simple and efficient method for CRISPR/Cas9-induced mutant screening. J Genet Genomics.

[61] Lamport D. Preparation of arabinogalactan glycoproteins from plant tissue. BIO-Protoc. 2013;3. 10.21769/BioProtoc.918.

[62] ØBro J, Harholt J, Scheller HV, Orfila C (2004). Rhamnogalacturonan I in Solanum tuberosum tubers contains complex arabinogalactan structures. Phytochemistry..

