# Supplementary material for: Elucidating the roles of three β-glucuronosyltransferases (GLCATs) acting on arabinogalactan-proteins using a CRISPR-Cas9 multiplexing approach in Arabidopsis
Source: BMC Plant Biol. 2020 May 18;20:221. doi: 10.1186/s12870-020-02420-5 (PMC7236193; doi:10.1186/s12870-020-02420-5)
Supplement: Supplementary file 1 — Additional file 1: Five supplemental tables, three supplemental figures and their corresponding legends. Table S1. List of guide RNA sequences and their target genes. Table S2. List of primers used to assemble three gRNAs (A1, B1, and C1) into pHEE401E binary vector. Table S3. List of primers used to assemble four gRNAs (A2, B1, C2, and C3) into pHEE401E binary vector. Table S4. List of primers for sequencing off-targets. Table S5. List of primers used for qRT-PCR. Figure S1. Amino acid sequence alignment of GLCAT14A, GLCAT14B, and GLCAT14C. Figure S2. Expression profiles of GLCAT14A, GLCAT14B, and GLCAT14C obtained from the Klepikova Arabidopsis Atlas eFP Browser of the Bio-Analytic Resource for Plant Biology (BAR) [18]. The link for the expression profile of GLCAT14A can be found at http://bar.utoronto.ca/efp/cgi-bin/efpWeb.cgi?primaryGene=AT5G39990&dataSource=Klepikova_Atlas&modeInput=Absolute; The link for the expression profile of GLCAT14B can be found at http://bar.utoronto.ca/efp/cgi-bin/efpWeb.cgi?primaryGene=AT5G15050&dataSource=Klepikova_Atlas&modeInput=Absolute; The link for the expression profile of GLCAT14C can be found at http://bar.utoronto.ca/efp/cgi-bin/efpWeb.cgi?primaryGene=AT2G37585&dataSource=Klepikova_Atlas&modeInput=Absolute. Figure S3. Expression patterns of GLCAT14A, GLCAT14B, and GLCAT14C during seed development obtained from the Arabidopsis eFP Browser of the Bio-Analytic Resource for Plant Biology (BAR) (bar.utoronto.ca). The link for the expression profile of GLCAT14A during seed development can be found at http://bar.utoronto.ca/efp/cgi-bin/efpWeb.cgi?dataSource=Seed&modeInput=Absolute&primaryGene=At5g39990&secondaryGene=At3g27340&override=None&threshold=219.05&modeMask_low=None&modeMask_stddev=None; The link for the expression profile of GLCAT14B during seed development can be found at http://bar.utoronto.ca/efp/cgi-bin/efpWeb.cgi?dataSource=Seed&modeInput=Absolute&primaryGene=AT5G15050&secondaryGene=At3g27340&override=&threshold=17.75 [file 12870_2020_2420_MOESM1_ESM.zip › Additional Files.docx]

**Additional Files**

**Additional File 1. Five supplemental tables, three supplemental figures and their corresponding legends.**

**Supplemental Table 1.** List of guide RNA sequences and their target genes.

**Supplemental Table 2.** List of primers used to assemble three gRNAs (A1, B1, and C1) into pHEE401E binary vector.

**Supplemental Table 3.** List of primers used to assemble four gRNAs (A2, B1, C2, and C3) into pHEE401E binary vector.

**Supplemental Table 4.** List of primers for sequencing off-targets.

**Supplemental Table 5.** List of primers used for qRT-PCR.

**Supplemental Figures**

**Supplemental Figure 1.** Amino acid sequence alignment of GLCAT14A, GLCAT14B, and GLCAT14C.

**Supplemental Figure 2.** Expression profiles of *GLCAT14A*, *GLCAT14B*, and *GLCAT14C* obtained from the Klepikova Arabidopsis Atlas eFP Browser of the Bio-Analytic Resource for Plant Biology (BAR) [18]. The link for the expression profile of *GLCAT14A* can be found at <http://bar.utoronto.ca/efp/cgi-bin/efpWeb.cgi?primaryGene=AT5G39990&dataSource=Klepikova_Atlas&modeInput=Absolute>; The link for the expression profile of *GLCAT14B* can be found at <http://bar.utoronto.ca/efp/cgi-bin/efpWeb.cgi?primaryGene=AT5G15050&dataSource=Klepikova_Atlas&modeInput=Absolute>; The link for the expression profile of *GLCAT14C* can be found at <http://bar.utoronto.ca/efp/cgi-bin/efpWeb.cgi?primaryGene=AT2G37585&dataSource=Klepikova_Atlas&modeInput=Absolute>

**Supplemental Figure 3.** Expression patterns of *GLCAT14A*, *GLCAT14B*, and *GLCAT14C* during seed development obtained from the Arabidopsis eFP Browser of the Bio-Analytic Resource for Plant Biology (BAR) (bar.utoronto.ca). The link for the expression profile of *GLCAT14A* during seed development can be found at <http://bar.utoronto.ca/efp/cgi-bin/efpWeb.cgi?dataSource=Seed&modeInput=Absolute&primaryGene=AT5G39990&secondaryGene=At3g27340&override=&threshold=25.18&modeMask_low=None&modeMask_stddev=None&gene_alias1=GlcAT14A&gene_alias2=>; The link for the expression profile of *GLCAT14B* during seed development can be found at <http://bar.utoronto.ca/efp/cgi-bin/efpWeb.cgi?dataSource=Seed&modeInput=Absolute&primaryGene=AT5G15050&secondaryGene=At3g27340&override=&threshold=17.75&modeMask_low=None&modeMask_stddev=None&gene_alias1=GlcAT14B&gene_alias2=>; The link for the expression profile of *GLCAT14C* during seed development can be found at <http://bar.utoronto.ca/efp/cgi-bin/efpWeb.cgi?dataSource=Seed&modeInput=Absolute&primaryGene=AT2G37585&secondaryGene=At3g27340&override=&threshold=18.13&modeMask_low=None&modeMask_stddev=None&gene_alias1=GlcAT14C&gene_alias2=>

**Supplemental Table 1.** List of guide RNA sequences and their target genes.

| **Target site (Target gene)** | **gRNA sequence** |
| --- | --- |
| A1 (*GLCAT14A*) | TCAATGAAGTTCAAATCCCG**CGG** |
| A2 (*GLCAT14A*) | GCAACATTGTTTCTTCAGAG**AGG** |
| B1 (*GLCAT14B*) | CTTGGATTCGACGAAGACGG**AGG** |
| C1 (*GLCAT14C*) | ACAGCGCACCCTGAATGTAA**CGG** |
| C2 (*GLCAT14C*) | GCTGGTCGTGAATAAGAACG**AGG** |
| C3 (*GLCAT14C*) | GAGATGGGAAACGAGTGAAG**CGG** |

**Supplemental Table 2.** List of primers used to assemble three gRNAs (A1, B1, and C1) into pHEE401E binary vector.

| **Purpose** | **Sequence** |
| --- | --- |
| **Cloning** | |
| DT1-A1_F | ATATATGGTCTCGATTGCAATGAAGTTCAAATCCCGGTT |
| DT1-A1_F0 | TGCAATGAAGTTCAAATCCCGGTTTTAGAGCTAGAAATAGC |
| DT0-R2 | ATATTATTGGTCTCAATCTCTTAGTCGACTCTACCAAT |
| DT2-B1_F | ATATTATTGGTCTCAAGATTGTTGGATTCGACGAAGACGGGTT |
| DT2-B1_F0 | TGTTGGATTCGACGAAGACGGGTTTTAGAGCTAGAAATAGC |
| DT3-C1_R0 | AACTTACATTCAGGGTGCGCTGCAATCACTACTTCGTCTCTAACCAT |
| DT3-C1_R | ATTATTGGTCTCGAAACTTACATTCAGGGTGCGCTGC |
| **Colony PCR** |  |
| U6-29p-F | TTAATCCAAACTACTGCAGCCTGAC |
| U6-1p-R | TATGCAAGTCTCACTCACACTCACG |
| **Sequencing** |  |
| U6-29p-R | AGCCCTCTTCTTTCGATCCATCAAC |
| U6-29p-F | TTAATCCAAACTACTGCAGCCTGAC |
| U6-29t-F | CGTGTTTCAGCTCTCATGATCCTTG |

**Supplemental Table 3.** List of primers used to assemble four gRNAs (A2, B1, C2, and C3) into pHEE401E binary vector.

| **Purpose** | **Sequence** |
| --- | --- |
| **Cloning** | |
| DT1-C3_F | ATATATGGTCTCGATTGAGATGGGAAACGAGTGAAGGTT |
| DT1-C3_F0 | TGAGATGGGAAACGAGTGAAGGTTTTAGAGCTAGAAATAGC |
| DT0-R2 | ATATTATTGGTCTCAATCTCTTAGTCGACTCTACCAAT |
| DT2-A2_F2 | ATATTATTGGTCTCAAGATTGCAACATTGTTTCTTCAGAGGTT |
| DT2-A2_F0 | TGCAACATTGTTTCTTCAGAGGTTTTAGAGCTAGAAATAGC |
| DT0-BsR3 | ATATTATTGGTCTCATCACTACTTCGTCTCTAACCAT |
| DT3-B1-BsF3 | ATATTATTGGTCTCAGTGATTGTTGGATTCGACGAAGACGGGTT |
| DT3-B1-F0 | TGTTGGATTCGACGAAGACGGGTTTTAGAGCTAGAAATAGC |
| DT4-C2-R0 | AACCGTTCTTATTCACGACCAGCAATCACTACTTCGACTCTAGC  TGTAT |
| DT4-C2-BsR | ATTATTGGTCTCGAAACTTACATTCAGGGTGCGCTGC |
| **Colony PCR** |  |
| U6-29p-F | TTAATCCAAACTACTGCAGCCTGAC |
| U6-1t-R | AACGGACCAATCACTTTGTCTTAGC |
| **Sequencing** |  |
| U6-29p-F | TTAATCCAAACTACTGCAGCCTGAC |
| U6-29p-R | AGCCCTCTTCTTTCGATCCATCAAC |
| U6-1t-F | GCTAAGACAAAGTGATTGGTCCGTT |
| U6-1t-R | AACGGACCAATCACTTTGTCTTAGC |

**Supplemental Table 4.** List of primers for sequencing off-targets.

| **Primer Name** | **Sequence** |
| --- | --- |
| A1_At5g58003_F | GAAGCATCCTCAAGCAAAGG |
| A1_At5g58003_R | CGGAAACATTGCAACCATCT |
| B1_At5g04160_F | CCAGGATTTCACTGGTTTGG |
| B1_At5g04160_R | GGCTGAATAAGCCATGACAGA |
| C1_At4g14640_F | ACCTTGAGCTCCCTCCTCTC |
| C1_At4g14640_R | TTAAAGGATTCCAAACAACCA |
| C1_At5g63420_F | AAGAACGGAGAAATGCTAGGAG |
| C1_At5g63420_R | TGTTTTCTGAGACCCCTGGA |

**Supplemental Table 5.** List of primers used for qRT-PCR.

| **Primers** | **Sequence** |
| --- | --- |
| 14A_F | GCTTTTCACAGGCTCTGCTTG |
| 14A_R | TCCTCAGCGTTGCAGAGAAC |
| 14B_F | GCAACATTGGCTGGAAAGAGTC |
| 14B_R | ATCCACGCAGATCCTGTGAAG |
| 14C_F | GAGCACACAAGCAACATCGG |
| 14C_R | ACGCTGGTTGAACCCATGAA |
