# Supplementary material for: Elucidating the roles of three β-glucuronosyltransferases (GLCATs) acting on arabinogalactan-proteins using a CRISPR-Cas9 multiplexing approach in Arabidopsis
Source: BMC Plant Biol. 2020 May 18;20:221. doi: 10.1186/s12870-020-02420-5 (PMC7236193; doi:10.1186/s12870-020-02420-5)

Supplemental Figure 1

```
AtGLCAT14A  115 LRRTLLALYHPNNRYVVHLDRESSREERREELHGYIKNSSLFRRFMNVHMIEKANLVTYRG
AtGLCAT14B  102 LKRTLMALYHPNNQYVVHLDRESSPEERLDLSGFVANHTLFQRFQNVRMIVKANFVTYRG
AtGLCAT14C   73 VKRLLKAIHHPRNYYLLHLDLEASDEERMELAKYVRSE--KKKFENVMVMGLADLVTEKG

AtGLCAT14A  175 PTMVANTLHAAAILLREGADWDWFINLSSSDYPLVTQDDLLHIFSHLPRDLNFIDHTSNI
AtGLCAT14B  162 PTMVANTLHAAAILLREGGDWDWFINLSASDYPLVTQDDLLHTFSYLPRDLNFIDHTSNI
AtGLCAT14C  131 PTMLASTLHGVAILLKKAKDWDWFINLSASDYPLMPQDDILHIFSYLPRYLNFIEHTSNI

AtGLCAT14A  235 GWKASQRAKPVIIDPGLYLNKKSDVFWVTQRRSIPTAFKLFTGSAWMALSRPFVDYCIWG
AtGLCAT14B  222 GWKESHRAKPIIIDPGLYMSKKADVFWVSQKRSMPTAFKLFTGSAWMMLSRPFVDYFIWG
AtGLCAT14C  191 GWKENQRARPIIIDPGFYHLKKSGVFWAKERRSLPASFKLFMGSTSVALTRPFLEFCIWG

AtGLCAT14A  295 WDNLPRTVLMYYSNFLSSPEGYFHTVLCNAEEFRNTTVNSDLHFISWDNPPKQHPHHLTL
AtGLCAT14B  282 WDNLPRIVLMYYANFLSSPEGYFHTVICNAREFTNTTVNSDLHFISWDNPPKQHPHHLTL
AtGLCAT14C  251 WDNLPRTLLMYYTNFLLSSEGYFQTVVCNNKDYQNTTVNHDLHYTKWD-PLQQRTLNVTV

AtGlcAT14A  355 TDMTKMVNSNAPFARKFRREDPVLDKIDDELLNRGPGMITPGGWCIGSHENGSDPCAVIG
AtGlcAT14B  342 DDFQRMVDSNAPFARKFRRDEPVLDKIDSELLFRSHGMVTPGGWCIGTRENGSDPCAVIG
AtGlcAT14C  310 ENFRDMVQSGAPFAREFREDDLVLDKIDIELLGQ------------------TDTGLELK

AtGLCAT14A  415 DTDVIRPGPGARRLENLVTSLLSTENFRSKQCK
AtGLCAT14B  402 DTSVIKPGLGAKRIEKLITYLLSTENFRPRQCR
AtGLCAT14C  352 TPDVVKPTVSWKRLEKLMVRLLDHENFRAKQCK
```

Supplemental Figure 2

GLCAT14A

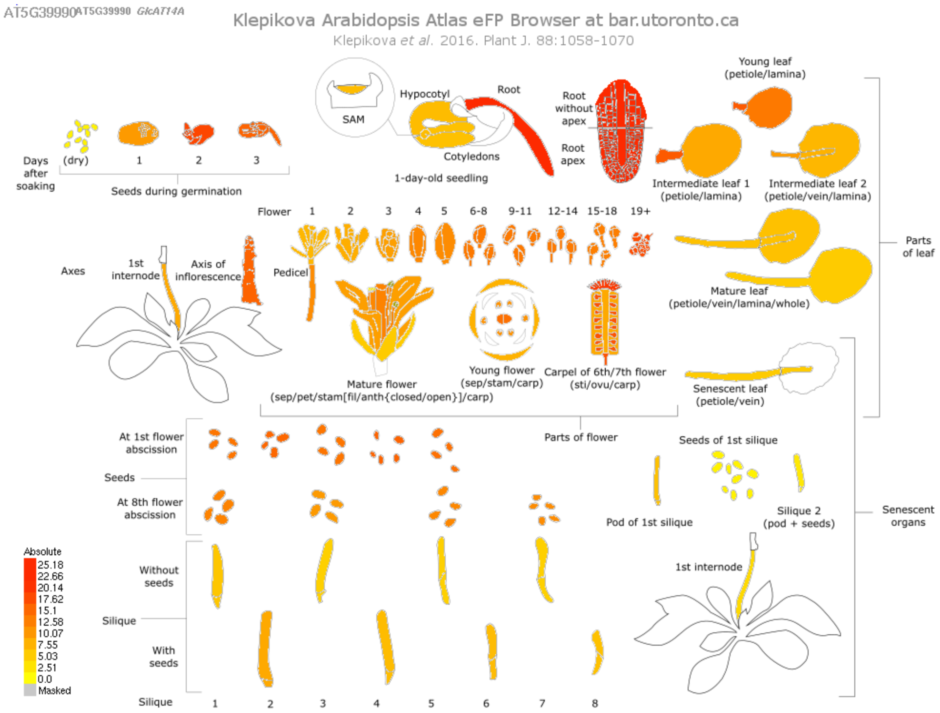

GLCAT14B

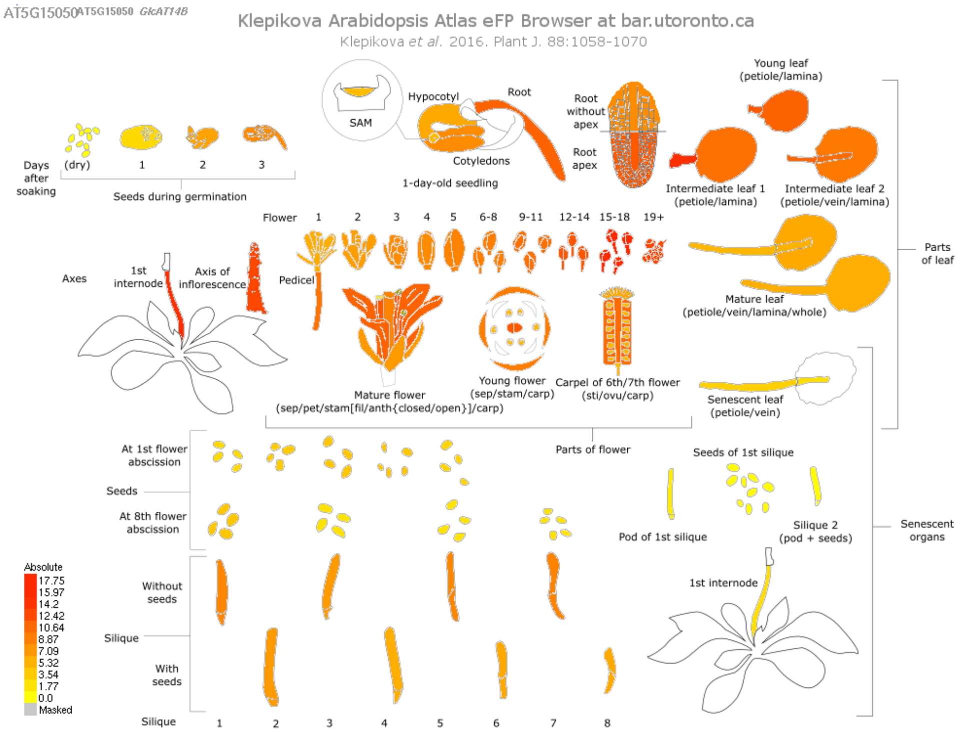

AT5G15050 AT5G15050 GlcAT14B
Klepikova Arabidopsis Atlas eFP Browser at bar.utoronto.ca
Klepikova et al. 2016. Plant J. 88:1058-1070
SAM
Hypocotyl
Root
Cotyledons
1-day-old seedling
Root without apex
Root apex
Young leaf (petiole/lamina)
Intermediate leaf 1 (petiole/lamina)
Intermediate leaf 2 (petiole/vein/lamina)
Days after soaking
(dry) 1 2 3
Seeds during germination
Flower 1 2 3 4 5 6-8 9-11 12-14 15-18 19+
Axes
1st internode
Axis of inflorescence
Pedicel
Mature flower (sep/pet/stam[fil/anth{closed/open}]/carp)
Young flower (sep/stam/carp)
Carpel of 6th/7th flower (sti/ovu/carp)
Mature leaf (petiole/vein/lamina/whole)
Senescent leaf (petiole/vein)
Parts of leaf
Parts of flower
At 1st flower abscission
Seeds
At 8th flower abscission
Seeds of 1st silique
Pod of 1st silique
Silique 2 (pod + seeds)
Senescent organs
1st internode
Absolute
17.75
15.97
14.2
12.42
10.64
8.87
7.09
5.32
3.54
1.77
0.0
Masked
Without seeds
Silique
With seeds
Silique 1 2 3 4 5 6 7 8

GLCAT14C

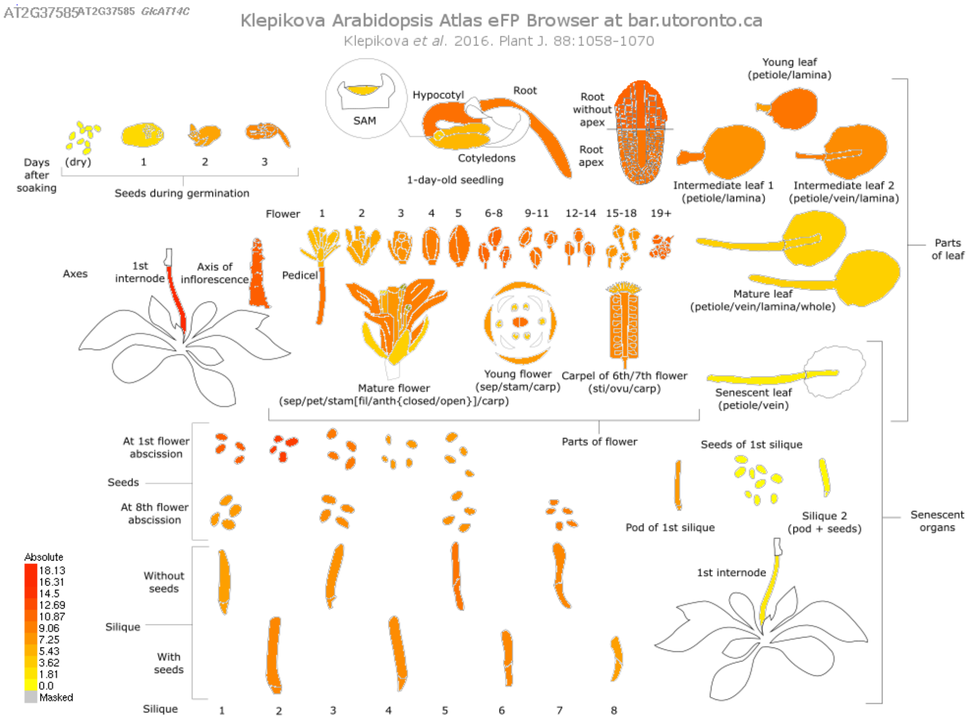
AT2G37585AT2G37585 GlcAT14C
Klepikova Arabidopsis Atlas eFP Browser at bar.utoronto.ca
Klepikova et al. 2016. Plant J. 88:1058-1070
SAM
Hypocotyl
Root
Cotyledons
1-day-old seedling
Root without apex
Root apex
Young leaf (petiole/lamina)
Intermediate leaf 1 (petiole/lamina)
Intermediate leaf 2 (petiole/vein/lamina)
Days after soaking
(dry)
1
2
3
Seeds during germination
Flower
1
2
3
4
5
6-8
9-11
12-14
15-18
19+
Axes
1st internode
Axis of inflorescence
Pedicel
Mature flower (sep/pet/stam[fil/anth{closed/open}]/carp)
Young flower (sep/stam/carp)
Carpel of 6th/7th flower (sti/ovu/carp)
Mature leaf (petiole/vein/lamina/whole)
Parts of leaf
Senescent leaf (petiole/vein)
Parts of flower
At 1st flower abscission
Seeds
At 8th flower abscission
Seeds of 1st silique
Pod of 1st silique
Silique 2 (pod + seeds)
Senescent organs
Absolute
18.13
16.31
14.5
12.69
10.87
9.06
7.25
5.43
3.62
1.81
0.0
Masked
Without seeds
Silique
With seeds
1st internode
Silique
1
2
3
4
5
6
7
8

Supplemental Figure 3

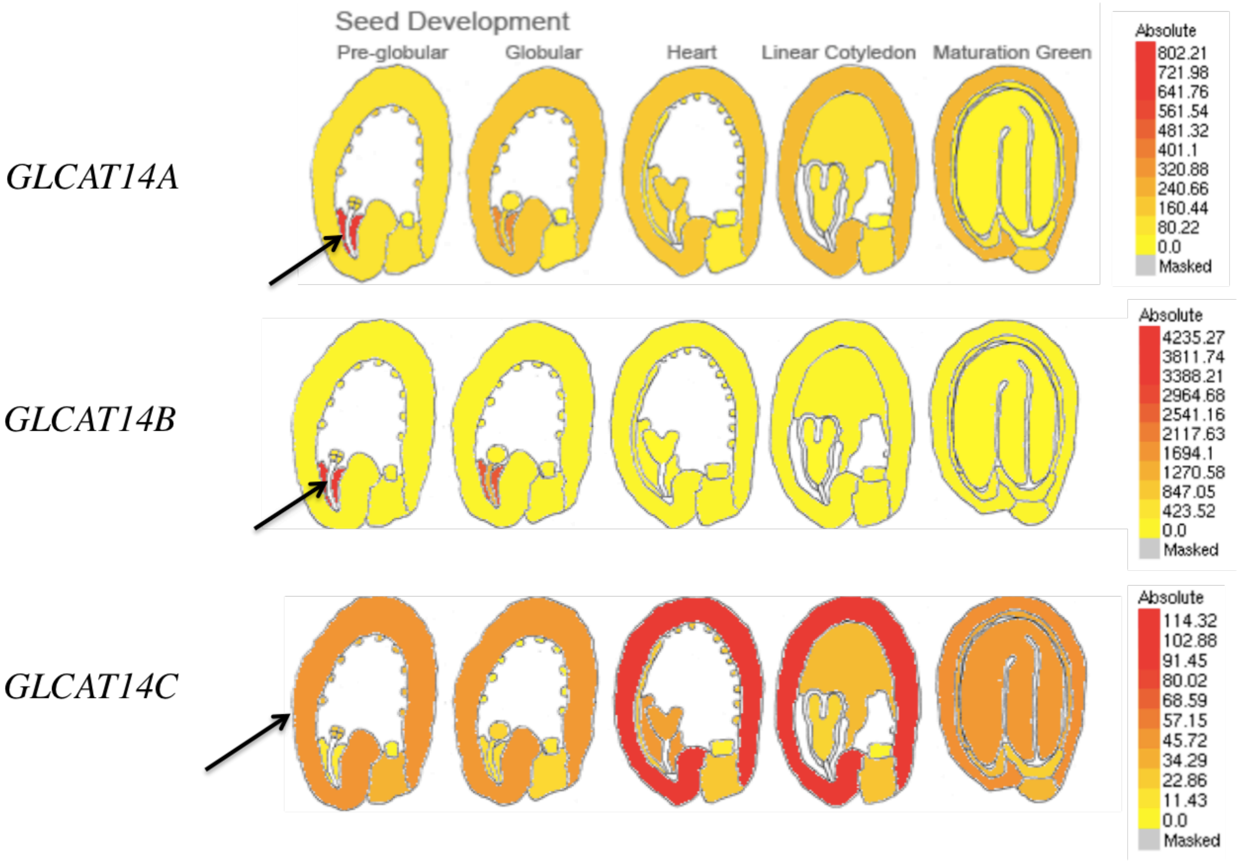

Supplement: Supplementary file 1 — Additional file 1: Five supplemental tables, three supplemental figures and their corresponding legends. Table S1. List of guide RNA sequences and their target genes. Table S2. List of primers used to assemble three gRNAs (A1, B1, and C1) into pHEE401E binary vector. Table S3. List of primers used to assemble four gRNAs (A2, B1, C2, and C3) into pHEE401E binary vector. Table S4. List of primers for sequencing off-targets. Table S5. List of primers used for qRT-PCR. Figure S1. Amino acid sequence alignment of GLCAT14A, GLCAT14B, and GLCAT14C. Figure S2. Expression profiles of GLCAT14A, GLCAT14B, and GLCAT14C obtained from the Klepikova Arabidopsis Atlas eFP Browser of the Bio-Analytic Resource for Plant Biology (BAR) [18]. The link for the expression profile of GLCAT14A can be found at http://bar.utoronto.ca/efp/cgi-bin/efpWeb.cgi?primaryGene=AT5G39990&dataSource=Klepikova_Atlas&modeInput=Absolute; The link for the expression profile of GLCAT14B can be found at http://bar.utoronto.ca/efp/cgi-bin/efpWeb.cgi?primaryGene=AT5G15050&dataSource=Klepikova_Atlas&modeInput=Absolute; The link for the expression profile of GLCAT14C can be found at http://bar.utoronto.ca/efp/cgi-bin/efpWeb.cgi?primaryGene=AT2G37585&dataSource=Klepikova_Atlas&modeInput=Absolute. Figure S3. Expression patterns of GLCAT14A, GLCAT14B, and GLCAT14C during seed development obtained from the Arabidopsis eFP Browser of the Bio-Analytic Resource for Plant Biology (BAR) (bar.utoronto.ca). The link for the expression profile of GLCAT14A during seed development can be found at http://bar.utoronto.ca/efp/cgi-bin/efpWeb.cgi?dataSource=Seed&modeInput=Absolute&primaryGene=At5g39990&secondaryGene=At3g27340&override=None&threshold=219.05&modeMask_low=None&modeMask_stddev=None; The link for the expression profile of GLCAT14B during seed development can be found at http://bar.utoronto.ca/efp/cgi-bin/efpWeb.cgi?dataSource=Seed&modeInput=Absolute&primaryGene=AT5G15050&secondaryGene=At3g27340&override=&threshold=17.75 [file 12870_2020_2420_MOESM1_ESM.zip › BMC Supplemental Fig.pdf]
